# A survey on generative adversarial networks for imbalance problems in computer vision tasks

**DOI:** 10.1186/s40537-021-00414-0

**Published:** 2021-01-29

**Authors:** Vignesh Sampath, Iñaki Maurtua, Juan José Aguilar Martín, Aitor Gutierrez

**Affiliations:** 1 Autonomous and Intelligent Systems Unit, Tekniker, Member of Basque Research and Technology Alliance, Eibar, Spain; 2 Design and Manufacturing Engineering Department, Universidad de Zaragoza, 3 María de Luna Street, Torres Quevedo Bld, 50018 Zaragoza, Spain

**Keywords:** Generative adversarial neural networks, Imbalanced data, Object detection, Segmentation, Classification, Deep learning, Deep generative model

## Abstract

Any computer vision application development starts off by acquiring images and data, then preprocessing and pattern recognition steps to perform a task. When the acquired images are highly imbalanced and not adequate, the desired task may not be achievable. Unfortunately, the occurrence of imbalance problems in acquired image datasets in certain complex real-world problems such as anomaly detection, emotion recognition, medical image analysis, fraud detection, metallic surface defect detection, disaster prediction, etc., are inevitable. The performance of computer vision algorithms can significantly deteriorate when the training dataset is imbalanced. In recent years, Generative Adversarial Neural Networks (GANs) have gained immense attention by researchers across a variety of application domains due to their capability to model complex real-world image data. It is particularly important that GANs can not only be used to generate synthetic images, but also its fascinating adversarial learning idea showed good potential in restoring balance in imbalanced datasets.

In this paper, we examine the most recent developments of GANs based techniques for addressing imbalance problems in image data. The real-world challenges and implementations of synthetic image generation based on GANs are extensively covered in this survey. Our survey first introduces various imbalance problems in computer vision tasks and its existing solutions, and then examines key concepts such as deep generative image models and GANs. After that, we propose a taxonomy to summarize GANs based techniques for addressing imbalance problems in computer vision tasks into three major categories: 1. Image level imbalances in classification, 2. object level imbalances in object detection and 3. pixel level imbalances in segmentation tasks. We elaborate the imbalance problems of each group, and provide GANs based solutions in each group. Readers will understand how GANs based techniques can handle the problem of imbalances and boost performance of the computer vision algorithms.

## Introduction

Recent developments in Convolutional Neural Networks (ConvNets) have led to substantial progress in the performance of computer vision tasks applied across various domains such as self-driving cars [1], medical imaging [2], agriculture [3, 4], manufacturing [5], etc. The availability of big data [6], together with increased computing capabilities is the predominant reason for the recent success. Image acquisition is the first step in the development of computer vision algorithms. When the acquired image is not adequate, the desired task may not be possible to achieve. Image classification [7], object detection [8] and segmentation [9] are the fundamental building blocks of the computer vision tasks. All these methods use deep ConvNets with enormous layers and have a very high number of parameters that need to be tuned. Therefore, they demand a huge amount of representative data to improve their performance and generalization ability. While the amount of visual data is increasing exponentially, many of the real-world datasets suffer from several forms of imbalance. Handling imbalances in the image dataset is one of the pervasive challenges in the field of computer vision.

Image classification is the task of classifying an input image according to a set of possible classes. Classification algorithms learn to isolate important distinguishing information about an object in an image like shape or color and ignore irrelevant parts of an image such as plane background or noise. Several popular image classification architectures such as LeNet [7], AlexNet [10], VGG-16 [11], GoogLeNet [12], ResNet [13], Inception-V3 [14], DenseNet [15] take an input image and then pass it through several convolutional and pooling layers. Convolutional layer helps to extract features from the input image, while a pooling layer reduces the dimension. Several successive convolutional and pooling layers may follow, depending on the layout and intent of the architecture. The result is a set of feature maps reduced in size from the original image that through a training process have learned to distill information about the content in the original image. All extracted feature maps are then transformed into a single vector that can be fed into a series of fully connected neural network to obtain a probability distribution of class scores. The predicted class for the input image can be extracted from this probability distribution.

**Fig. 1:**
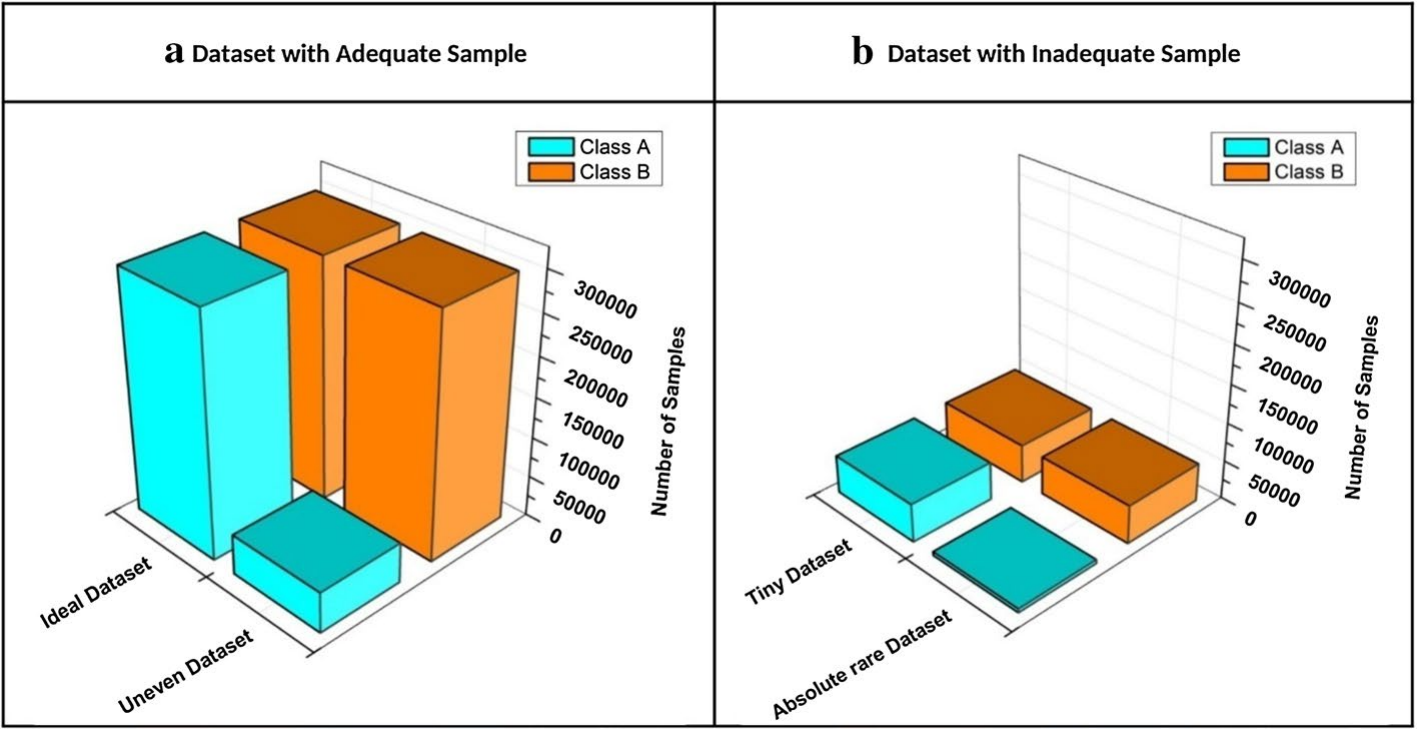
Distribution of different type of datasets (**a**) Dataset with adequate sample (**b**) Dataset with inadequate sample

These architectures are typically designed to work well with balanced datasets, but a common issue with real-world datasets is the imbalance of observed classes. The most commonly known imbalance problem in a task of image classification is the class imbalance. Class imbalance in the real-world image datasets is ubiquitous and can have an adverse effect on the performance of ConvNets [16]. These datasets usually fall into four categories in terms of its size and imbalance [17]:

1. The ideal datasets are the one that contain an adequate and equal or almost equal number of samples within each class. An equal probability is assigned to all classes during training to update parameters of the network and approach the minimum value of the error function. A wide range of standard machine learning algorithms can be applied for the ideal datasets.
2. The datasets with an adequate number of samples where some instances of classes are rarer than other instances of classes are said to be uneven datasets. Even though these datasets have adequate number of samples, it is costly and may not be possible for experts to manually inspect huge unlabeled datasets to annotate.
3. Tiny datasets are not easily available, and they can be difficult to collect. Such datasets have an equal number of samples within each class, but they are almost impossible to collect due to privacy restriction and other reasons.
4. Absolute rare datasets have a limited number of samples and substantial class imbalance. Reasons for class imbalance in these datasets can vary but commonly the problem arises because of: (a) Very limited number of experts available for data collection; for an example, generation of medical imaging datasets requires specialized equipment and well trained medical practitioners for data acquisition (b) Enormous manual effort required to label datasets; and (c) Scarcity of samples of specific class leading to class imbalance. Consequently, the size of the dataset and class imbalance problem becomes a bottleneck that prevents us from tapping the true potential of ConvNets. Figure 1 illustrates different types of datasets in terms of its size and imbalance.

Class imbalance in a dataset can stem from either between classes (inter class imbalance) or within class (intra class imbalance). Inter class imbalance occurs when a minority class contains a smaller number of instances when compared to instances belonging to the majority class. Classifiers built using inter class imbalanced datasets are most likely to predict minority class as rare occurrences, even sometimes assumed as outlier or noise which results in misclassification of minority classes [18]. Minority classes are often of greater interest and significance, that needs to be cautiously handled. For example, in a rare disease medical diagnosis where there is a vital need to distinguish such a rare medical condition among the normal populations. Any kind of diagnosis errors will cause stress to the patient and further complications. It is therefore very important that deep learning models [19] built using such datasets should be able to achieve a higher detection rate on minority classes.

Intra class imbalance in a dataset can also deteriorate the performance of the classifier. An Intra-class imbalance can be viewed as the attribute bias within a class, in other words inter-class imbalance in fine-grained visual categorization. For example, a class of dog samples can be further categorized by dog color, pose variations and dog breeds. Imbalances in such categories (intra class imbalance) is an unavoidable problem in datasets of many classification tasks such as modality based medical image classification [19], fine grained attribute classification [20], person re-identification [21], age [22] and pose invariant face recognition [23].

Several attempts have been made to overcome the problem of class imbalance by using different approaches and techniques. These techniques can be grouped into data-level approaches, algorithm level methods and hybrid techniques. While data level approaches modify the distribution of training set to restore balance by adding or removing instances from the training dataset, algorithm level methods change the objective function of the classifier to increase the importance of the minority class. Hybrid techniques combine algorithm level methods with data level approaches. Next few paragraphs will inform readers about some of the traditional techniques available to counter the class imbalance problem.

- *Resampling* To counteract the class imbalance problem, two types of re-sampling can be applied: One is under sampling by deleting samples from the majority class and another is oversampling by duplicating samples from the minority class [24]. Re-sampling method balances the dataset but fails to provide any additional information to the training set. The other limitations of this method include: oversampling results in over fitting problem while under sampling leads to substantial loss of information [25]. The quantity of under-sampling and oversampling is generally determined using experimental methods and empirically established [26]. In order to yield additional information to the training set, synthetic oversampling methods create new samples instead of duplicates to add equilibrium to skewed distribution. The Synthetic Minority Oversampling Technique (SMOTE) [27] is a popular synthetic oversampling method that aims to generate synthetic samples based on randomly selected K-nearest neighbors. SMOTE does not take account of the distribution of data between the classes. Adaptive synthetic sampling (ADASYN) approach [28] uses a weighted distribution for different minority classes according to their learning difficulties to adaptively generate synthetic data samples. Cluster based oversampling [29] technique divides the input space into various clusters and then incorporates sampling to alter the sample size. Many traditional synthetic oversampling techniques such as SMOTE or ADASYN are only suitable for low dimensional tabular data which restricts their application in a high dimensional image data. In addition, all the aforementioned techniques generate data by either deleting or averaging existing data, and hence may fail to improve classification performance.
- *Augmentative oversampling* Data augmentation is another commonly used technique to inflate the size of the training dataset [30]. Augmentation such as translation, cropping, padding, rotation and horizontal flipping introduces small modifications in the image data, but not all these modifications will improve the performance of a classifier. There is no standard method that can decide whether any particular augmentation strategy can improve results until the training process is complete. As training ConvNets is a time-consuming process [31], only a restricted amount of augmentation strategy is likely to be tested before model deployment. Also, the diversity that can be obtained from small modifications of the images is relatively small. In addition to balancing classes by oversampling, augmentation techniques also serve as a kind of regularization in deep neural network architecture and hence reduce the chance of over fitting. There is no consensus about the best strategy for combining different augmentation strategies together. Therefore, more advanced augmentation techniques such as mixing images depend on expert knowledge for validation and labelling [32]. A complete survey of Image data augmentation for deep learning has been compiled by Shorten et al. [32].
- *Semi-supervised learning (SSL)* SSL [33] is one of the most attractive ways to improve classification performance where we have access to small number of labeled samples ${\text{x}}$ ${\text{x}}$ along with large amount of unlabeled samples (Uneven dataset). SSL uses the combination of supervised and unsupervised learning techniques. It makes use of small labeled samples as the training set to train the model in a supervised manner, and then use the trained model to predict on the remaining unlabeled portion of the dataset. The process of labeling each sample of unlabeled data with the individual outputs predicted for them using the trained model is known as pseudo labeling. After labeling the unlabeled data through the pseudo labeling process, classification model is trained on both the actual and pseudo labeled data. Pseudo labeling is an interesting paradigm to annotate large-scale unlabeled data that potentially takes many tedious hours of human labor to manually label them. However, SSL relies on assumptions about the underlying marginal distribution of input data $p\left( x \right)$, both the labeled and unlabeled samples are assumed to have the same marginal distribution. This marginal distribution $p\left( x \right)$ should contain information about the posterior distribution $p(y|x)$. A complete list of semi supervised learning is detailed in [34].
- *Cost sensitive learning* Majority of the classification algorithms assume that misclassification costs of both minority and majority classes are the same. Cost-sensitive learning [35] pays more attention to misclassification costs of the minority class through a cost matrix.

The most straightforward and commonly used approach in ConvNets is the data driven strategy, because deep ConvNets with enormous layers have a very high number of parameters to be tuned, it is prone to overfitting when trained on a small sized dataset. Data level approaches inflate the training data size that serves as regularization and hence reduce the chance of overfitting in deep neural network architecture. Traditional data-level techniques suffer the following drawbacks, particularly when used for the class imbalance problem in high-dimensional image data.

- Synthetic instances created using traditional data level approaches may not be the true representative of the training set.
- Synthetic data generation is achieved either by duplication or linear interpolation which does not generate new examples that are atypical and puzzle the classifier decision boundaries, and hence fail to improve overall performance.
- In Medical images, augmentation techniques are restricted to minor alteration on an image, as they abide by strict standards. Additionally, the types of augmentation one can use vary from problem to problem. For instance, heavy augmentations such as geometric transformations, random erasing, and mixing images might damage semantic content of the medical image.
- Applying data augmentation in an absolute rare dataset may not provide the variations required to produce a distinct sample to add equilibrium to skewed distribution.
- Dealing with the class imbalance in fine-grained visual categorization is challenging because it involves large intra-class variability and small inter-class variability.
- Most of the techniques are designed only for binary classification problems. Multi class imbalance problems are generally considered much harder than their binary equivalents for many reasons. For Instance, there can be several combinations of minority-majority classes, i.e., they may include: 1. Few minority-Many majority classes, 2. Many minority-Few majority classes, and 3. Many minority-Many majority classes.

Class imbalance in image classification tasks has been widely explored and studied. In addition to class imbalance, there are many different forms of imbalances that can impede performance of other computer vision tasks such as object detection and image segmentation. Object detection, which deals with localization and classification of multiple objects in a given image, is another challenging and significant task in computer vision. The typical way of localizing an object in an image is by drawing a bounding box around the object. This bounding box can be interpreted as a collection of coordinates that define the box. Nowadays, object detection algorithms fall into two broad categories: two-stage detectors and single stage detectors. On one hand, two stage detector such as Region-based Convolutional Neural Networks (R-CNN) [8], Fast R-CNN [36], Faster R-CNN [37], Mask R-CNN [38], etc. employ a Region Proposal Network (RPN) to search objects in the first stage, and then process these region of interests for object classification and bounding-box regression in the second stage. On the other hand, single stage detectors such as Single Shot Detection (SSD) [39], You Only Look Once (YOLO) [40], etc. perform detection on a grid that avoids spending too much time on generating region proposals. Instead of locating objects perfectly, they prioritize speed and recognition. Therefore, one stage object detectors are fast and simple, whereas two stage detectors are more accurate.

Despite the recent advances, applying object detection algorithms to the real-world datasets such as in-car video [41], transportation surveillance images [42] that contain objects with large variance of scales (Objects scale imbalance) remains challenging. Physical size of a same object at different distances from the camera would appear as different size. Singh et al. [43] showed that object level scale variation greatly affects the overall performance of object detectors. Many solutions have been proposed to address the object scale imbalance. Scale aware fast R-CNN [44] uses an ensemble of two object detectors, one for detecting the large and medium scale objects and other for the small scale objects, and then combines them to produce final predictions. Multi-scale Image Pyramids such as SNIP [43] and SNIPER [45] use an image pyramid to build multi scale feature representation. Feature Pyramid Networks (FPN) [46] combine feature hierarchies at different scales to predict objects at different scales.

Objects in the real-world datasets only occupy a small portion of the image, while the rest of the image is background. Both single and two stage algorithms approximately evaluate about 10^4^ to 10^5^ locations per image [47], yet just a few locations have objects. The imbalance between foreground (object) and background can also hinder performance of the object detection algorithm. Furthermore, object detection algorithms should be invariant to deformation and occluded objects. In Pedestrian detection Dataset [48], for instance, more than 70% of pedestrians are occluded in at least one frame of a video clip and about 19% of pedestrians are occluded in all frames, where the occlusions are ranked as heavy in almost half of such cases. Dollar et al. [48] highlight that the performance of pedestrian detection using standard detectors declines substantially even under partial occlusion, and drastically under severe occlusion. Data augmentation based on random erasing [49] is a frequently used technique that forces detectors to pay attention to the entire object in an image, rather than just a portion of it. Yet, this technique is not guaranteed to be advantageous in all the conditions. Because skewed distributions arise even within deformed and occluded objects as some of the occlusions and deformations are uncommon that they hardly occur in practical scenarios [50].

Image segmentation that classifies every pixel in an image suffers from pixel level imbalances, as are other computer vision tasks.Some of the well-known image segmentation algorithms include Fully connected network [9], SegNet [51], U-Net [52], ResUNet [53] etc. Image segmentation is essential for a variety of tasks, including: Urban scene segmentation for autonomous driving [54], industrial inspection [55] and cancer cell segmentation [56]. Datasets of all these tasks suffer from pixel level imbalance. For example, In Urban street scene dataset [57], Pixels corresponding to sky, building and road are far numerous than pixels of pedestrian and bicyclist. This is due to the fact that the area covered by sky, buildings and roads are more than pedestrians and bicyclists in the image. Similarly, In brain tumour image segmentation dataset [58], MRI images have more healthy brain tissue pixels than cancerous tissue pixels. The most frequently used loss function for image segmentation task is a pixel wise cross entropy loss [59]. This loss assigns equal weights to all the pixels, evaluates the prediction for each pixel individually and then averages over all pixels. In order to mitigate this problem, many works have been done which modify the pixel wise cross entropy loss function. The standard cross entropy loss is modified in Weighted cross entropy [52], Focal loss [47], Dice Loss [60], Generalised Dice Loss [61], Tversky loss [62], Lovász-Softmax [63] and Median frequency balancing [51], so as to assign higher importance to rare pixels. Although modified loss functions are efficient for some imbalances, such functions undergo severe difficulties when it comes to highly imbalanced datasets, as seen with medical image segmentations.

In contrast to all the traditional approaches described above, Generative adversarial Neural Networks (GANs) aim to learn underlying true data distributions from the limited available images (both minority and majority class), and then use the learned distributions to generate synthetic images. This raises an interesting question on whether GANs can be used to generate synthetic images for the minority class of various imbalanced datasets. Indeed, recent developments of GANs suggest that being capable to represent complex and high dimensional data can be used as a method of intelligent oversampling. GANs utilize the ability of neural networks to learn a function that can approximate model distribution as close as possible to true distribution. Particularly, they do not rely on prior assumptions about the data distribution and can generate synthetic images with high visual fidelity. This significant property allows GANs to be applied to any kind of imbalance problem in computer vision tasks. GANs can not only be able to generate a fake image, but also offer a way to change something about the original image. In other words, they can learn to produce any desired number of classes (such as, objects, identities, people, etc.), and across many variations (such as, viewpoints, light conditions, scale, backgrounds, and more). There are a wide variety of GANs reported in the literature, each with their own strengths to alleviate imbalance problem in computer vision tasks. For instance, AttGAN [64], IcGAN [65], ResAttr-GAN [66], etc. are a specific variant of GANs that are commonly used for facial attribute editing tasks. They learn to synthesize not only a new face image with desired attributes but also preserves attribute independent details. Recently, GANs have been combined with a wide range of existing object detection and image segmentation algorithms to overcome the problem of imbalance and improve their performance.

The original GANs architecture [67] contains two differentiable functions represented by two networks, a generator $G$ and a discriminator $D$. The learning procedure of GANs is to simultaneously train a discriminator $D$ and a generator $G$. It follows an adversarial two-player, zero-sum game. An intuitive way of understanding GAN is with the police and the counterfeiter anecdote. The generator network is like a group of counterfeiters trying to produce fake money and make it look genuine. The police attempt to discover counterfeiters using fake money, yet at the same time need to let every other person spend their real money. Over time, the police show signs of improvement at identifying fake cash, and the forgers improve at faking it. In the end, the counterfeiters are compelled to make ideal copies of real money. High resolution and realistic minority class images generated using learned model distribution can be used to balance the class distribution and mitigating effect of over fitting by inflating the training dataset size. GANs solve the problem of generating data when there is not enough data to begin with and they require no human supervision. GANs can provide an efficient way to fill in holes in the discrete distribution of training data. In other words, they can transform the discrete distribution of training data to continuous, providing an additional data by nonlinear interpolation between the discrete points. Bowles et al. [68] argues that GANs offer an access to unlock additional information from a dataset. In fact, Yann LeCun, the facebook vice president and chief AI scientist, referred to GANs as "*the most interesting thing that has happened to the field of machine learning in the last 10 years*".

In this survey, as opposed to other related surveys on class imbalance, that present class imbalance in tabular data, we focus on wide range of imbalance in high dimensional image data by following a systematic approach with a view to help researchers establish a detailed understanding of GAN based synthetic image generation for the imbalance problems in computer vision tasks. Furthermore, our survey covers imbalances in a wide range of computer vision tasks in contrast to other surveys that are limited to image classification tasks.

**Fig. 2:**
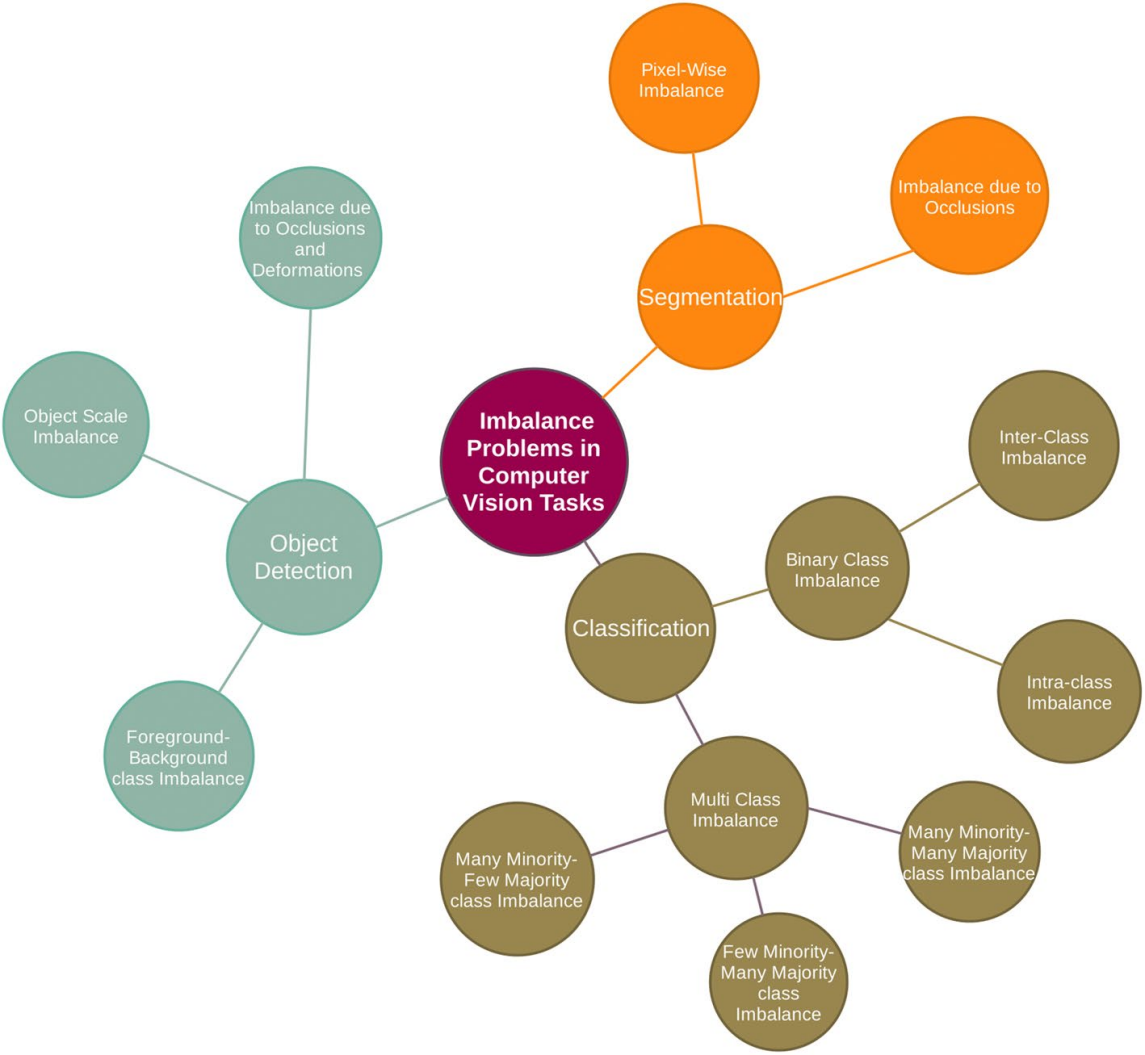
Proposed taxonomy for the review of imbalanced problem in computer vision tasks

The key contributions of this survey are presented as follows:

- In this survey paper, we review current research work on GAN based synthetic image generation for the imbalance problems in visual recognition tasks spanning from 2014 to 2020. We group these imbalance problems in a taxonomic tree with three main groups: Classification, Object detection and Segmentation (Fig. 2).
- Also, we provide necessary material to inform research communities about the latest development and essential technical components in the field of GAN based synthetic image generation.
- Apart from analyzing different GAN architectures, our survey focuses heavily on real world applications where GAN based synthetic images are used to alleviate imbalances and fills a research gap in the use of synthetic images for the imbalance problems in visual recognition tasks.

The remainder of this paper is organized as follows: “Deep Generative image models” section gives readers necessary background information on generative models. “Generative adversarial Neural Network” section discusses selected GAN variants from the architecture, algorithm, and training tricks perspective in detail. In “Taxonomy of class imbalance in visual recognition tasks” section, we provide a brief explanation on various types of imbalances encountered in visual recognition tasks and how the GAN based synthetic image is used to rebalance, followed by GAN variants from the application perspective. “Discussion and Future work” section identifies and enumerates our perspective and possible future research direction. Finally, we conclude the paper in “Conclusion” section.

## Deep generative image models

Deep Generative model is an important family of unsupervised learning methods that are dedicated to describe the underlying distribution of unlabeled training data and learn to generate brand new data from that distribution. Color image data [32] is pixel values encoded into a three-dimensional stacked array, made up of height, width, and three-color channels. Modeling the distribution of image data is extremely challenging as natural images are high dimensional and highly structured [69]. This challenge has led to a rich variety of neural network based generative image models, each having their own advantages. Research into neural network based generative models for image generation has a long history. Restricted Boltzmann Machines [70–72] and their deep variants [73–75] are a popular class of probabilistic models for image generation. Now the generative image models can be grouped into three broad categories: 1. Autoregressive models, 2. Latent variable models and 3. Adversarial learning-based models.

*Autoregressive models (ARs)* aim to estimate a distribution over images (density estimation) using a joint distribution of the pixels in the image by casting it as a product of conditional distributions [76]. ARs transform the problem of joint modeling into a sequence problem, where, given all the pixels previously generated, one learns to predict the next pixel. But a highly powerful sequence model is needed to model the highly non-linear and long span auto correlations between the pixels. Based on this idea, many research articles have been published that use different sequence models from deep learning to model the complex conditional distribution. Fully visible belief network (FVBN) [77, 78] is one of the tractable explicit density models that use chain rule to factorize likelihood of an image ${\text{x}}$ into product of one dimension distributions, where ${\text{n}} \times {\text{n}}$. pixels in the greyscale image is taken row by row as a one dimensional sequence ${\text{x}}_{1} ,{\text{x}}_{2} ,{\text{x}}_{3} \ldots ,{\text{x}}_{{{\text{n}}^{2} }}$. The joint likelihood ${\text{p}}\left( {\text{x}} \right)$ is explicitly computed as the product of the conditional probabilities over the pixels. The conditional distribution of each pixel in an image is calculated as shown in Eq. (1).

1 $$p\left( x \right) = { }\mathop \prod \limits_{j = 1}^{{n^{2} }} p(x_{j} |x_{1} ,x_{2} ,...x_{j - 1} )$$

ven all the preceding pixels $x_{1} ,x_{2} \ldots x_{j - 1}$, the value $p(x_{j} |x_{1} ,x_{2} ,...x_{j - 1} )$ is the probability of the j-th pixel $x_{j}$. Each pixel is dependent on previous pixels that have been already generated. The pixel generation starts from the corner, continues pixel by pixel and row by row. In the case of an RGB image, each pixel value in an individual RGB color is jointly computed by three values, one for each of the RGB color channels. The conditional distribution $p(x_{j} |X < j)$ can be rewritten as the following product (Eq. (2)) where green channel is conditioned on channel red and blue channel is conditioned on channels red and green.

2 $$p(x_{j} ,R|X < j)p(x_{j} ,G|X < j,x_{j} ,R)p(x_{j} ,B|X < j,x_{j} ,R,x_{j} ,G)$$

**Fig. 3:**
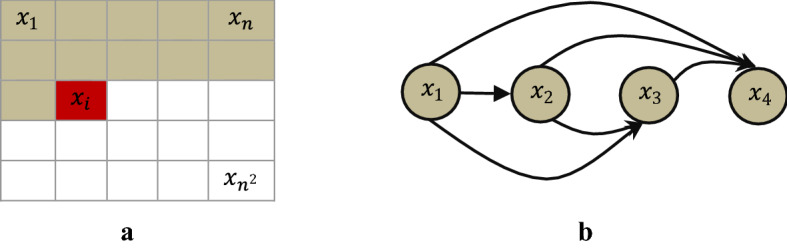
Autoregressive models train a network that models conditional distribution of each pixel given all previous pixels. The image is processed pixel-by-pixel in (**a**) Raster scan order and (**b**) Sequentially predicts pixels

Generating an image pixel by pixel using this approach is sequential, computationally intense, and a very slow process as each of the colour channels is conditioned on the other channels as well as on all the pixels generated previously (Fig. 3).

Neural Autoregressive Density Estimator (NADE) [79] aims to learn a joint distribution using a neural network to parametrize the factors of ${\text{p}}\left( {\text{x}} \right)$. The output layer of the NADE is designed to predict n conditional probability distributions, each node in the output layer corresponds to one of the factors in the joint distribution. Hidden representation for each output node is computed using only relevant inputs, i.e. only previous ${\text{i}} - 1$ input variables are connected to the ith output. By implementing a neural network, NADE allows weights sharing that reduces the number of parameters to learn a joint distribution using stochastic gradient descent.

Recurrent neural networks (RNN) have been proved to excel at various sequential tasks, such as speech recognition [80], speech synthesis [81], handwriting recognition [82], and image to text [83]. Particularly, Long Short-Term Memory (LSTM) layers [84], transformers and self-attention mechanism [85] are the robust architecture for modeling long range sequence data with auto correlations like time series data, natural languages etc. In order to have a long-term memory, LSTM layer adds gates to the RNN. It has an input to state component and a recurrent state to state component that together determine the gates of the layer. Theis et al. [86] used spatial LSTM (sLSTM), a multi-dimensional LSTM which is suitable for image modeling because of its spatial structure. However, an immense amount of time is needed to train the LSTM layers considering the number of pixels in the larger datasets such as CIFAR-10 [87] and ImageNet [88].

Van den Oord et al. [69] designed two variants of recurrent image models: PixelRNN and PixelCNN. The pixel distributions of the natural images are modeled with two-dimensional LSTM (spatial LSTMs) and convolutional networks in PixelRNN and PixelCNN respectively. Convolution operation enables PixelCNNs to generate pixels faster than PixelRNNs, given the large number pixels in natural images. But typically, PixelRNNs achieve higher performance when compared to PixelCNNs. Gated PixelCNN [89] is another interesting paradigm to generate diverse natural images with a density model conditioned on prior information along with previously generated pixels. The prior information ${\text{h}}$ in Eq. (4) can be any vector, including class labels or tags.

3 $$p\left( {x|h} \right) = \mathop \prod \limits_{j = 1}^{{n^{2} }} p(x_{j} |x_{1} ,x_{2} ,...x_{j - 1} ,h)$$

A lot of work on improving performance of PixelCNN has been reported in literature by introducing new architectures, loss functions and different training tricks. PixelCNN +  + [90] enhances the performance of PixelCNN by proposing numerous modifications while retaining its computational performance. Major modifications include: 1. Intensity of a pixel is viewed as 8-bit discrete random variables and modeled using 256-softmax output in pixelCNN. In contrast, PixelCNN +  + uses discretized logistic mixture likelihood to model each pixel as real valued output. 2. It simplifies the model structure by conditioning on entire pixels, instead of RGB sub space. 3. PixelCNN +  + employs down-sampling by using convolution of stride 2 in order to capture structure at multiple resolutions 4.Short cut connections are added to compensate the loss of information due to down-sampling. 5. PixelCNN +  + also introduces model regularization using dropouts. Pixel Snail [91] incorporates a self-attention mechanism in PixelCNN to have access to long term temporal information.

*Latent variable models* on the other hand, aim to represent high dimensional image data (observable variables) into lower dimensional latent space (latent variables). Latent variables as opposed to observable variables are variables that are not directly observed but inferred through a model from other variables that are observed directly. One advantage of using latent variable is that it reduces dimensionality of data. High dimensional observable variables can be aggregated in a model to represent an underlying concept making it easier to understand the data.

Autoencoders are one of the latent variable models that take unlabeled high dimensional image data $x$, after encoding them into lower dimensional feature representation $z$, try to reconstruct them as accurately as possible. The lower dimensional feature $z$ is a compressed representation of an input image, as a result, the autoencoder must decide which of the features in an image are the most important, essentially acting as a feature extraction engine or dimensionality reduction. They are typically very shallow neural networks, and usually consist of an input layer, an output layer, and a hidden layer. Autoencoders with nonlinear encoder and decoder functions learn to project image data onto a nonlinear manifold, which are capable of performing powerful nonlinear generalization compared to principle component analysis (PCA). They are trained with back-propagation, using a metric called Reconstruction loss. Reconstruction loss measures the amount of information that was lost when an autoencoder tried to reconstruct the input, using pixel wise L1 or L2 distance. In other words, pixel wise distance between original images $x$ and reconstructed images $\hat{x}$. Autoencoders with a small loss value can produce reconstructed images that look very similar to the original images.

Traditionally, autoencoders are used for data denoising, data compression and dimensionality reduction. There are many variants of autoencoder proposed in the literature [92–97]. Deep autoencoders [93] use a stack of layers as encoder and decoder instead of limiting to a single layer. Sparse autoencoders [94] have a larger number of hidden neurons than the input or output neurons, but only a fraction of hidden neurons are permitted to be active at once. ConvNets are used as encoder and decoder in convolutional autoencoders [98]. In order to learn a function that is robust to minor variations in its training dataset, contractive autoencoders [96] add a penalty term to its objective function. Denoising autoencoders [92] are stochastic forms of the basic autoencoder that add white noise to the training data to reduce a situation of learning the identity function.

**Fig. 4:**
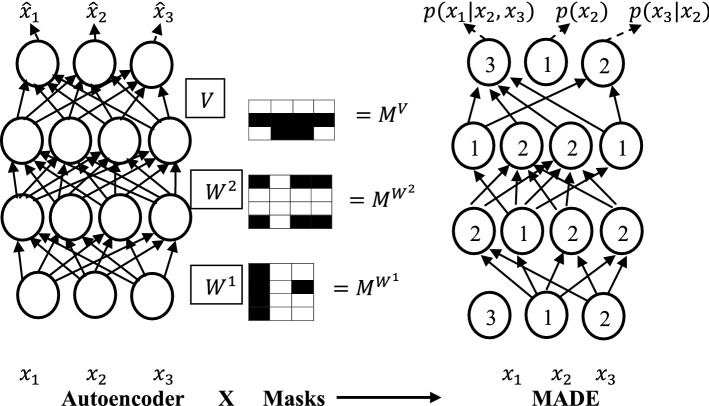
An illustration of Masked Autoencoder Density Estimator (MADE) [99]. A set of connections in an autoencoder is removed using multiplicative binary masks, such that each output unit is connected only to relevant input units

An autoencoder is tweaked to predict the $n$-conditional distributions rather than just reconstructing the inputs in Masked Autoencoder Density Estimator (MADE) [99]. In the standard fully connected autoencoder *i*th output unit depends on all the input units, but in order to predict the conditional distributions, *i*th output unit should depend only on previous $i - 1$ input variables. MADE modifies the autoencoder using a binary mask matrix to ensure each output unit is connected only to relevant input units (Fig. 4). As opposed to autoencoders that are used for an image abstraction, MADE is designed for image generation using learnt distribution (Fig. 4).

Variational Autoencoders (VAEs) [97] are the most popular class of autoencoders. In VAEs, the encoder instead of outputting a latent vector directly, outputs mean $\mu$ and variance $\sigma$ vectors which constitutes latent probability distributions $q_{\emptyset } \left( {z|x} \right)$ from which a latent vector is sampled. This means that given the same input image, no two latent vectors sampled are the same, which forces the decoder to learn the mapping from a region of a latent space to a reconstruction rather than just from a single point resulting in a much smoother reconstructed image. Unlike traditional autoencoders, which are only able to reconstruct images similar to training set, VAEs can generate new images close to training set. VAEs are trained by maximizing the variational lower bound (Eq. (4)) also known as evidence lower bound [100].

4 $${\mathcal{L}}_{VAE} \left( {\theta ,\emptyset ;x,z} \right) = \underbrace {{D_{KL} \left( {q_{\emptyset } \left( {z|x} \right)||p\left( z \right)} \right)}}_{Latent\ loss} - \underbrace {{E_{{z\sim q_{\emptyset } \left( {z{|}x} \right)}} \left( {logP_{\theta } \left( {x{|}z} \right)} \right)}}_{Reconstruction\ loss}$$

The first term in Eq. (4) is the Latent loss which regularizes the distribution of q to be Gaussian normal distribution ${\mathcal{N}}\left( {0,1} \right)$ by minimizing Kullback–Leibler divergence (KL divergence). KL divergence measures similarity between the latent probability distribution and the prior distribution using relative entropy. KL divergence from probability distribution q to p is defined to be

5 $$D_{KL} (q|{|}p{)} = \mathop \sum \limits_{x} q\left( x \right)log\frac{q\left( x \right)}{{p\left( x \right)}}$$

The latent loss is high when the latent probability distribution does not resemble a standard multivariate Gaussian and it is low when the resemblance between those two distributions is close. Given input data $x$, a probabilistic encoder encodes them to latent representation $z$ with distribution $q_{\emptyset } \left( {z|x} \right)$ and a probabilistic decoder decodes $p_{\theta } \left( {x|z} \right)$. Latent loss enforces the posterior distribution of latent representation $z$ to match with an arbitrary prior distribution $p\left( z \right)$. In other words, it imposes a restriction in $z$, such that input data $x$ are distributed in a latent space following a specified arbitrary prior distribution. The second term, reconstruction loss is pixel wise Binary cross entropy between original image $x$ and reconstructed image $\hat{x}$.

**Fig. 5:**
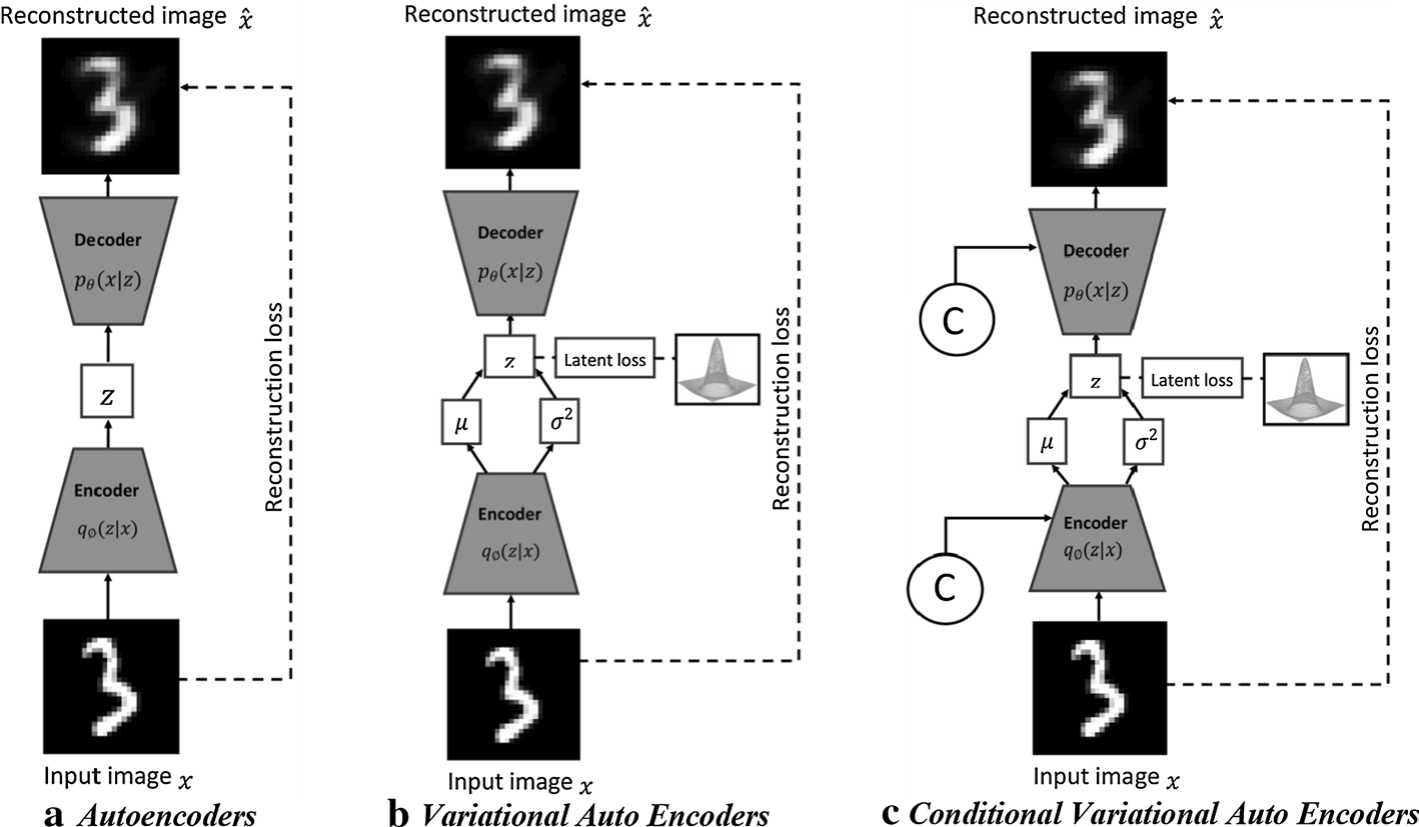
The architecture of (**a**) Autoencoders; **b** Variational Auto Encoders; **c** Conditional Variational Auto Encoders

The numerous modifications have been made over basic VAEs that was initially introduced in [97]. The Conditional VAE (CVAE) [101] is a conditioned version of standard VAEs (Fig. 5c) to generate diverse reconstructed images conditioned on additional information such as class labels, facial attributes etc. Variational lower bound of CVAE is written as

6 $${\mathcal{L}}_{CVAE} \left( {\theta ,\emptyset ;x,z,c} \right) = D_{KL} \left( {q_{\emptyset } \left( {z|x,c} \right)||p\left( {z,c} \right)} \right) - E_{{z\sim q_{\emptyset } \left( {z{|}x} \right)}} \left( {logP_{\theta } \left( {x{|}z,c} \right)} \right)$$

Beta VAE (β-VAE) [102] is another modified form of original VAE intended to learn disentangled latent representations that capture the independent features of a given image. It introduces additional hyper parameter $\beta$ that balances the latent and reconstruction loss. Variational lower bound of β-VAE is defined as

7 $${\mathcal{L}}_{\beta - VAE} \left( {\theta ,\emptyset ,\beta ;x,z} \right) = \beta [D_{KL} \left( {q_{\emptyset } \left( {z|x} \right)||p\left( z \right)} \right)] - E_{{z\sim q_{\emptyset } \left( {z{|}x} \right)}} \left( {logP_{\theta } \left( {x{|}z} \right)} \right)$$

When $\beta = 1$ in Eq. (7), it corresponds to the standard VAE framework. β-VAE with $\beta > 1$ pushes the model to learn disentangled representation. Deep Convolutional Inverse Graphics Network (DC-IGN) [103] replaced feed forward neural networks in the encoder and decoder of VAEs with convolution and deconvolution operators respectively. Importance weighted VAE (IWVAE) [104] learns richer and more complex latent space representation than VAEs from importance weighting. Convolutional VAE is combined with the PixelCNN in PixelVAE [105] and Variational lossy autoencoder [106]. Deep Recurrent Attentive Writer (DRAW) [107] networks combine spatial attention mechanism with a sequential variational autoencoder. In order to avoid problems of posterior collapse, Vector Quantized VAE (VQ-VAEs) [108] learns discrete latent representation instead of continuous normal distribution. VQ-VAEs combine VAEs with ideas from vector quantization to get a sequence of discrete latent variables. VQ-VAE 2 [109] is a Hierarchical multi-scale VQ-VAE combined with a self-attention mechanism for generating high resolution images.

*Adversarial models* try to model the distribution of the real data through an adversarial process. Generative adversarial neural networks based on game theory, introduced by Goodfellow et al. [67] in 2014, is arguably one of the best innovations in recent years. The word adversarial in generative adversarial neural networks means that the two neural networks, the generator and the discriminator are in a competition with each other. The learning procedure of GAN is to simultaneously train a discriminator $D$ and a generator$G$. The generator network takes a noise vector $z$ in a latent space as an input, then runs that noise vector through a differentiable function to transform the noise vector $z$ to create a fake but plausible image $x$:$G\left( z \right) \to x$. At the same time, the discriminator network, which is essentially a binary classifier, tries to distinguish between the real images (label 1) and artificially generated images by generator network (label 0):$D\left( x \right) \to \left[ {0,1} \right]$. Therefore, the objective function of GANs can be defined as

8 $$\mathop {\min }\limits_{G} \mathop {\max }\limits_{D} V\left( {D,G} \right) = E_{{x\sim p_{r} \left( x \right)}} \left[ {logD\left( x \right)} \right] + E_{{z\sim p_{z} \left( z \right)}} \left[ {{\text{log}}\left( {1 - D\left( {G\left( z \right)} \right)} \right)} \right]$$

Given random noise vector $z$ and real image $x$, the generator attempts to minimize ${\text{log}}(1 - D\left( {G\left( z \right)} \right)$ and the discriminator attempts to maximize $logD\left( x \right)$ in Eq. (8). For fixed$G$, the optimal $D$ is given by

9 $$D^{*} \left( x \right) = \frac{{p_{r} \left( x \right)}}{{p_{g} \left( x \right) + p_{r} \left( x \right)}}$$

Theoretically, when $G$ is trained to its optimal, the generated data distribution $p_{g} \left( x \right)$ gets closer to the real data distribution $p_{r} \left( x \right)$. If $p_{g} \left( x \right) = p_{r} \left( x \right), D^{*} \left( x \right)$ in Eq. (9) becomes ½. This means that the discriminator is maximally puzzled and cannot distinguish fake images from real ones. When the discriminator $D$ is optimal, the loss function for the generator $G$ can be visualized by substituting in ${\text{D}}^{*} \left( {\text{x}} \right)$ Eq. (8).

10 $$\begin{aligned} G^{*} = & \mathop {\max }\limits_{D} V\left( {G,D^{*} } \right) = E_{{x\sim p_{r} \left( x \right)}} \left[ {logD^{*} \left( x \right)} \right] + E_{{x\sim p_{g} \left( x \right)}} \left[ {{\text{log}}\left( {1 - D^{*} \left( x \right)} \right)} \right] \\ \; = & \;E_{{x\sim p_{r} \left( x \right)}} \left[ {log\frac{{p_{r} \left( x \right)}}{{\frac{1}{2}[p_{g} \left( x \right) + p_{r} \left( x \right)]}}} \right] + E_{{x\sim p_{g} \left( x \right)}} \left[ {log\frac{{p_{g} \left( x \right)}}{{\frac{1}{2}[p_{g} \left( x \right) + p_{r} \left( x \right)]}}} \right] - 2log2 \\ \end{aligned}$$

The definition of Jensen-Shannon divergence ($D_{JS}$) between two probability distributions $p_{g} \left( x \right)$ and $p_{r} \left( x \right)$ is defined as

11 $$D_{JS} (p_{r} ||p_{g} ) = \frac{1}{2}D_{KL} (p_{r} ||\frac{{p_{r} + p_{g} }}{2}) + \frac{1}{2}D_{KL} (p_{g} ||\frac{{p_{r} + p_{g} }}{2})$$

Therefore, Eq. (10) is equal to

12 $$G^{*} = 2D_{JS} (p_{r} \left( x \right)||p_{g} \left( x \right)) - 2log2$$

Essentially, the loss for the generator $G$ minimizes the Jensen-Shannon divergence between the generated data distribution ${\text{p}}_{{\text{g}}} \left( {\text{x}} \right)$ and the real data distribution ${\text{p}}_{{\text{r}}} \left( {\text{x}} \right)$ when discriminator $D$ is optimal. Jensen-Shannon divergence is a smooth, symmetric version of the KL divergence. Huszar [110] believes that the main reason behind the great success of GANs is replacing asymmetric KL divergence loss function in the classical approach to symmetric JS divergence.

**Fig. 6:**
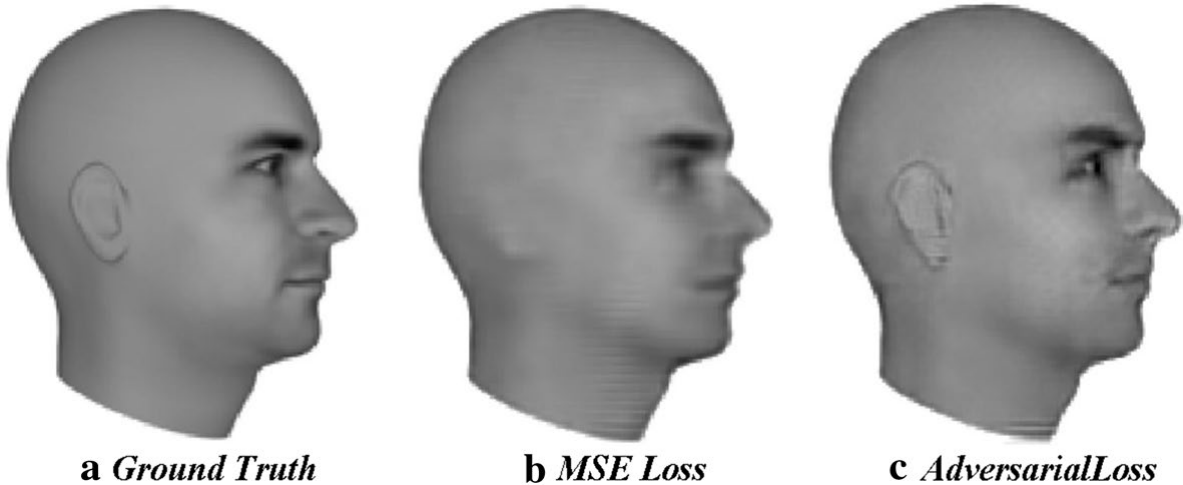
An illustration of the importance of an adversarial loss [111]

Mean squared error used in latent variable models such as autoencoder, averages all the possible features in an image and generate blurry images. In contrast, adversarial loss preserves the features using discriminator networks that detect an absence of any features as an unrealistic image. An example of this is the study carried out by Lotter et al. [111], in which models trained using mean square loss and adversarial loss to predict the next image frame in a video sequence are compared. A model trained using mean square loss generates blurry images as shown in Fig. 6, where ear and eyes are not sharply defined as they could be. Using an additional adversarial loss, features like the eyes and ear remain preserved very well, because an ear is the recognizable pattern, and the discriminator network would not accept any sample that is missing an ear.

**Fig. 7:**
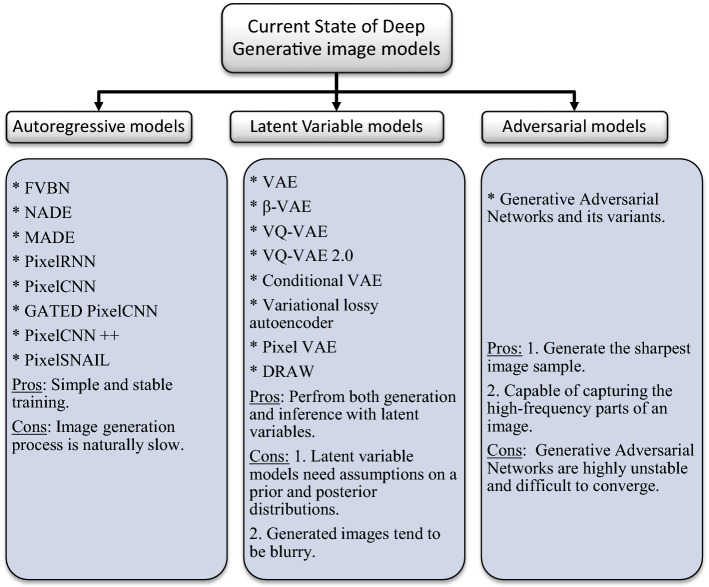
Comparative summary of Deep generative models discussed in “Deep Generative image models” section

This section has attempted to provide readers a brief introduction to the current state of deep generative image models. A quick summary of this section is depicted below in Fig. 7.

Despite remarkable achievements in generating sharp and realistic images, GANs suffer from certain drawbacks.

- *Non convergence* Both generator and discriminator networks in GANs are trained simultaneously using gradient descent in a zero-sum game. As a result, improvement of the generator network comes at the expense of discriminator and vice versa. Hence there is no guarantee of GANs convergence.
- *Mode collapse* Generator network achieves a state where it continues to generate samples with little variety, although trained on diverse datasets. This form of failure is referred to as mode collapse.
- *Vanishing gradient problems *If the discriminator is perfectly trained early in the training process, then there would be no gradients left to train the generator due to vanishing gradients.

Therefore, many GAN-variants have been proposed to overcome these drawbacks. These GAN-variants can be grouped into three categories:

1. *Architecture variants* In terms of architecture of generator and discriminator networks, the first proposed GANs use the Multi- layer perceptron (MLP). Owing to the fact that ConvNets work well with high resolution image data taking into account of the spatial structure of data, a Deep Convolutional GAN (DCGAN) [112] replaced the MLP with the deconvolutional and convolutional layers in generator and discriminator networks respectively.
  Autoencoder based GANs such as AAE [113], BiGAN [114], VAE-GAN [115], DEGAN [116], VEEGAN [117] etc., have been proposed to combine their construction power of autoencoders with the sampling power of GANs.
  Conditional based GANs like Conditional GAN (CGAN) [118], Auxiliary Classifier GAN (ACGAN) [119], VACGAN [120], infoGAN [121], and SCGAN [122] focused on controlling mode of data being generated by conditioning model on conditional variable.
2. *Training tricks* GANs are difficult to train. Improved trainings tricks such as feature matching, minibatch discrimination, historical averaging, one-sided label smoothing, and Two Time-Scale Update Rule have been suggested to ensure that GANs converge to achieve Nash equilibrium.
3. *Objective variants* In order to improve the stability and overcome vanishing gradient problems, different objective functions have been explored in [123–130].

The following section of this review moves on to describe in greater detail the selected GAN variants.

## Generative adversarial neural networks

### Architecture variants

**Fig. 8:**
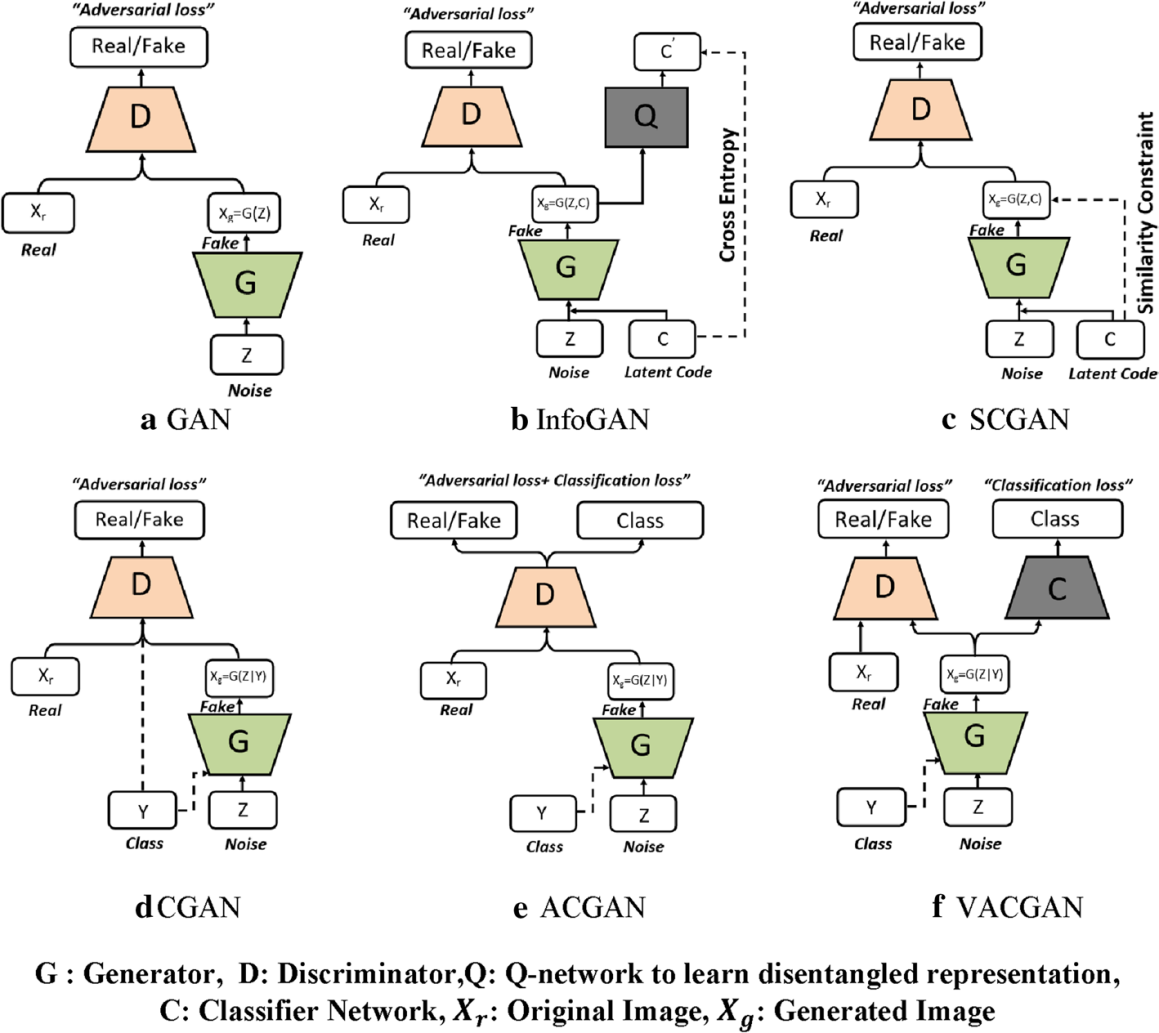
A schematic view of (**a**) the vanilla GAN and (**b–f**) variants of Conditional GANs

**Fig. 9:**
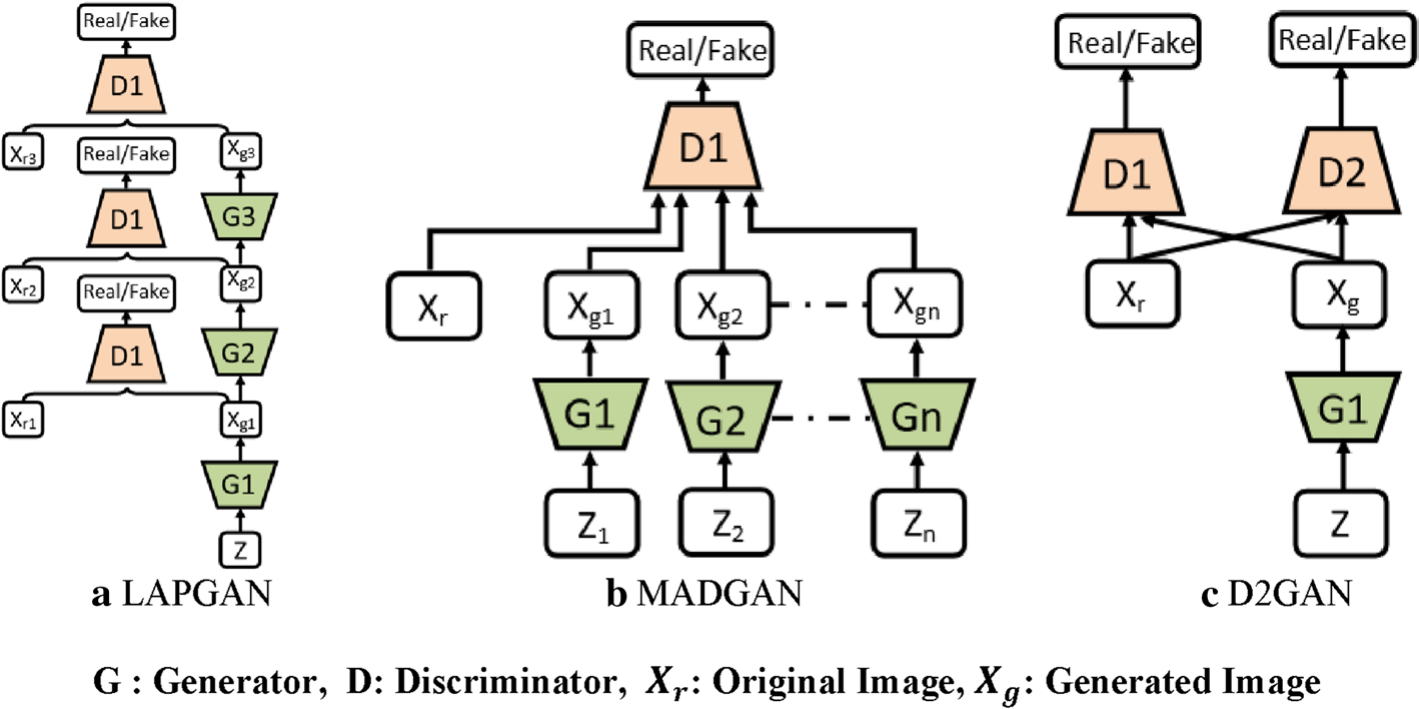
A schematic view of Variants of GANs with multiple discriminators and generators: **a** LAPGAN, **b** MADGAN and **c** D2GAN

**Fig. 10:**
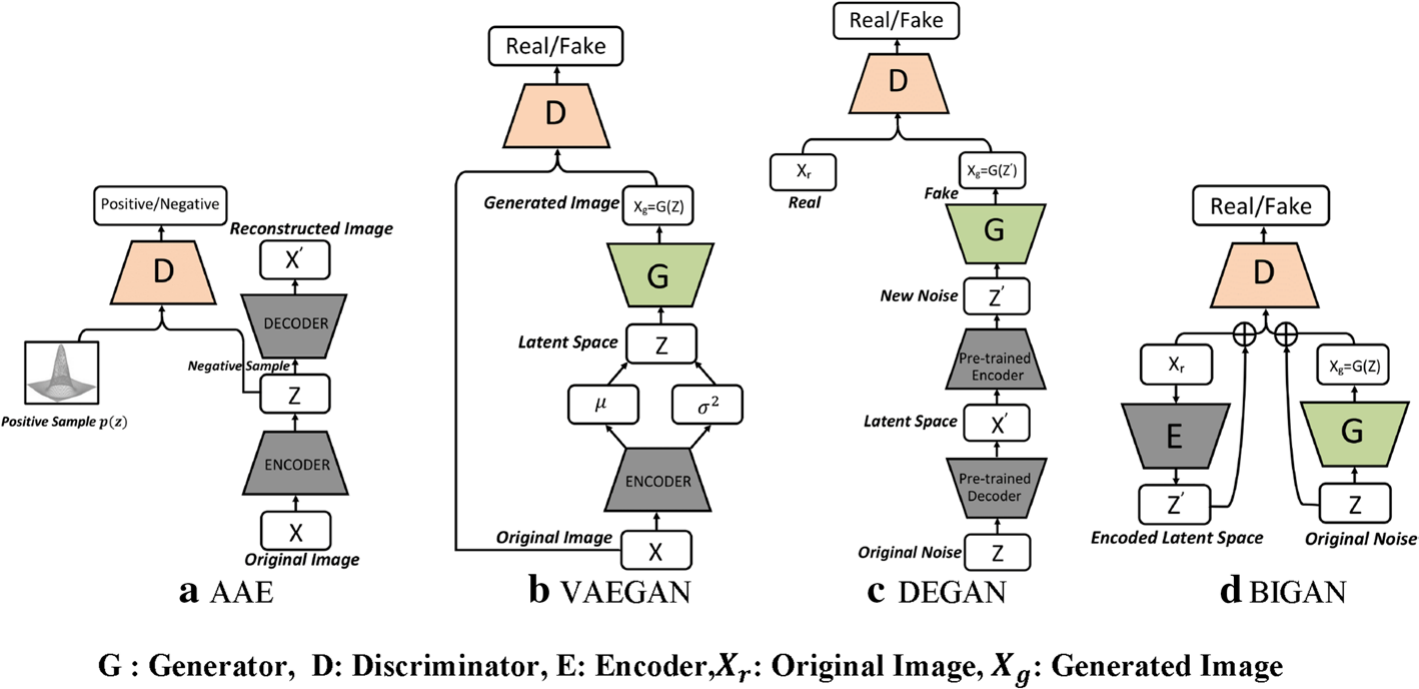
A schematic view of Variants of GANs based on Encoder and decoder architecture: **a** AAE, **b** VAEGAN, **c** DEGAN and **d** BIGAN

The performance and training stability of GANs are highly influenced by the architecture of the generator and the discriminator networks. Various architecture variants of GANs have been proposed that adopt several techniques to improve performance and stability.

i. *Conditional based GAN Variants*
  The standard GAN [67] architecture does not have any control on the modes of data being generated. Van den Oord et al. [89] argue that the class conditioned image generation can significantly enhance the quality of generated images. Several conditional based GANs have been proposed that learn to sample from a conditional distribution $p(x|y)$ instead of marginal $p\left( x \right).$ Conditional based GANs variants (Fig. 8) can be classified into two groups: 1. Supervised and 2. Unsupervised conditional GANs.
  Supervised conditional GANs variants require a pair of images and corresponding prior information such as class label. The prior information could be class labels, textual descriptions, or data from other modalities.
  *cGAN* Mirza and Osindero [118] proposed conditional Generative Adversarial Network (cGAN), to have a control on kind of data being generated by conditioning the model on prior information $y$. Both discriminator and generator in cGAN are conditioned by feeding $y$ as additional input. Using this prior information, cGAN is guided to generate output images with desired properties during the generation process.
  *ACGAN* Auxiliary classifier Generative Adversarial Network (ACGAN) [119] is an extension of the cGAN architecture. The discriminator in the ACGAN receives only the image, unlike the cGAN that gets both the image and the class label as input. It is modified to distinguish real and fake data as well as reconstruct class labels. Therefore, in addition to real fake discrimination, the discriminator also predicts class label of the image using an auxiliary decoder network.
  *VACGAN* The major problem with ACGAN is that it will affect the training convergence because of mixing the loss of classifier and discriminator into a single loss. Versatile Auxiliary Generative Adversarial Network (VACGAN) [120] separates out classifier loss by introducing a classifier network in parallel to the discriminator.
  No prior information is used in unsupervised conditional GAN variants to control on modes of the image being generated. Instead, feature information such as hair color, age, gender etc. is learned during the training process. Therefore, they need an additional algorithm to decompose the latent space into disentangled latent vector $c$, which contains the meaning features, and standard input noise vector z. The content and representation of an image is then controlled by noise vector z and disentangled latent vector $c$ respectively.
  *Info-GAN *Information maximizing Generative Adversarial Network (Info-GAN) [121] splits an input latent space into the standard noise vector $z$ and additional latent vector $c$. The latent vector $c$ is then made meaningful disentangled representation by maximizing the mutual information between latent vector $c$ and generated images $G\left( {z,c} \right)$ using additional Q network.
  *SC-GAN *Similarity constraint Generative Adversarial Network (SC-GAN) [122] attempts to learn disentangled latent representation by adding the similarity constraint between latent vector $c$ and generated images $G\left( {z,c} \right)$. Info-GAN uses an extra network to learn disentangle representation, while SC-GAN only adds an additional constraint to a standard GAN. Therefore, SCGAN simplifies the architecture of Info-GAN.
ii. *Convolutional based GAN*
  *DCGAN *Deep Convolutional Generative Adversarial Network (DCGAN) [112] is the first work that deploys convolutional and transpose-convolutional layers in the discriminator and generator, respectively. The salient features of the DCGAN architecture are enumerated as follows:
• First, the generator in DCGAN consists of fractional convolutional layers, batch normalization layers and ReLU activation functions.
• Second, the discriminator is composed of strided convolutional layers, batch normalization layers and Leaky ReLU activation functions.
• Third, uses Adaptive Moment Estimation (ADAM) optimizer instead of stochastic gradient descent with momentum.
iii. *Multiple GANs*
  In order to accomplish more than one goal, several frameworks extend the standard GAN to either multiple discriminators, generators, or both (Fig. 9).
  *ProGAN *In an attempt to synthesize higher resolution images Progressive Growing of Generative Adversarial Network (ProGAN) [131] stacks each layer of the generator and discriminator in a progressive manner as training progresses.
  *LAPGAN *Laplacian Generative Adversarial Network (LAPGAN) [132] is proposed for the generation of high quality images. This architecture uses a cascade of ConvNets within a Laplacian pyramid framework. LAPGAN utilizes several Generator-Discriminator networks at multiple levels of a Laplacian Pyramid for an image detail enhancement. Motivated by the success of sequential generation, Im et al. [133] introduced Generative Recurrent Adversarial Networks (GRAN) based on recurrent network that generate high quality images in a sequential process, rather than in one shot.
  *D2GAN *Dual discriminator Generative Adversarial Network (D2GAN) [134] employs two discriminators and one generator to address the problem of mode collapse. Unlike GANs, D2GAN formulates a three-player game that utilizes two discriminators to minimize the KL and reverse KL divergences between true data and the generated data distribution.
  *MADGAN* Multi-agent diverse Generative Adversarial Network (MADGAN) [135] incorporates multiple generators that discover diverse modes of the data while maintaining high quality of generated images. To ensure that different generators learn to generate images from different modes of the data, the objective of discriminator is modified to detect the generator which generated the given fake image along with discriminating the real and fake images.
  *CoGAN* Coupled GAN(CoGAN) [136] is used for generating pair of like images in two different domains. CoGAN is composed of a set of GANs–GAN1 and GAN2, each accountable for synthesizing images in one domain. It leans a joint distribution from two-domain images which are drawn individually from the marginal distributions.
  *CycleGAN and DiscoGAN* [137] use two generators and two discriminators to accomplish unpaired image to image translation tasks. CycleGAN [138] adopts the concept of cycle consistency from machine translation, where a sentence translated from English to Spanish and translate it back from Spanish to English should be identical.
iv. *Autoencoder based GAN Variants*
  The standard GANs architecture is unidirectional and can only map from latent space $z$ to data space $x$, while autoencoders are bidirectional. The latent space learned by encoders is the distribution that contains compressed representation of the real images. Several variants of GANs that combine GAN and encoder architecture are proposed to make use of the distribution learned by encoders (Fig. 10). Attributes editing of an image directly on data space $x$ is complex as image distributions are highly structured and high dimensional. Interpolation on latent space can facilitate to render complicated adjustments in the data space $x$.

*DEGAN* In standard GANs architecture, the input to the generator network is the noise vector that is randomly sampled from a Gaussian distribution $N\left( {0,1} \right)$, which may create a deviation from the true distribution of real images. Decoder Encoder Generative adversarial Network (DEGAN) [116] adopt decoder and encoder structure from VAE, pretrained on the real images. The pretrained decoder and encoder structure transform random Gaussian noise to distribution that contains intrinsic information of the images which is used as input of the generator network.

*VAEGAN* Variational autoencoder Generative Adversarial Network (VAEGAN) [115] jointly trains VAE and GAN by replacing the decoder of VAE with GAN framework. VAEGAN employs feature wise adversarial loss of GAN in lieu of element wise reconstruction loss of VAE to improve quality of image generated by VAE. In addition to latent loss and adversarial loss, VAEGAN uses content loss, also known as perceptual loss, which compares two images based on high level feature representation from pre-trained VGG Network [11].

*AAE* Unlike VAEGAN that discriminates in data space, adversarial autoencoders (AAE) [113] imposes a discriminator on the latent space as learning the latent code distribution is simpler than data distribution. The discriminator network discriminates between a sample drawn from latent space and from the distribution $p\left( z \right)$ that we are trying to model.

*ALI and BiGAN *In addition to generator network, Adversarially Learned Inference (ALI) [114] model and Bidirectional Generative Adversarial Network (BiGAN) contain an encoder component E that simultaneously learn inverse mapping of the input data $x$ to the latent code $z$. Unlike other variants of GAN where the discriminator network receives only real or artificially generated images, in the BiGAN and ALI model, the discriminator network receives both image and latent code pair.

*VEEGAN* [117]: addresses the problem of mode collapse through addition of a reconstruction network that reverses the action of the generator network. Reconstruction network takes in synthetic images then transforms them to noise, while generator network takes noise as an input and reconstructs them into synthetic image. In addition to adversarial loss, difference between the reconstructed noise and initial noise is used to train the network. Both generator and reconstruction networks are jointly trained, which encourages generator network to learn true distribution, hence solving the mode collapse problem.

Several other GANs have been proposed for image super resolution. The goal of super resolution is to upsample low resolution images to a high resolution one. Ledig et al. proposed Super-Resolution GAN (SRGAN) [139] for image super resolution,which takes poor quality image as input, and generates high quality image with 4 × resolution. The generator of the SRGAN uses very deep convolutional layers with residual blocks. In addition to an adversarial loss, SRGAN includes a content loss. The content loss is computed as the euclidean distance between the feature maps of the generated high quality image and the ground truth image, where feature maps are obtained from a pretrained VGG19 [140] network. Zhang et al. [141] combined a self attention mechanism with GANs (SAGAN) to handle long range dependencies that make the generated image look more globally coherent. Image-to-image translation GANs such as Pix2Pix GAN [142], Pix2pix HD GAN [143], and CycleGAN [137] learn to map an input image from a source domain to an output image from a target domain. A summary of architectural variants of GANs are summarized in Table 1.

**Table 1:** An overview of GANs variants discussed in “Architecture variants” section

Categories	GAN Type	Main Architectural Contributions to GAN
*Basic GAN*	GAN [67]	Use Multilayer perceptron in the generator and discriminator
*Convolutional Based GAN*	DCGAN [112]	Employ Convolutional and transpose-convolutional layers in the discriminator and generator respectively
	PROGAN [131]	Progressively grow layers of GAN as training progresses
*Condition based GANs*	cGAN [118]	Control kind of image being generated using prior information
	ACGAN [119]	Add a classifier loss in addition to adversarial loss to reconstruct class labels
	VACGAN [120]	Separate out classifier loss of ACGAN by introducing separate classifier network parallel to the discriminator
	infoGAN [121]	Learn disentangled latent representation by maximizing mutual information between latent vector and generated images
	SCGAN [122]	Learn disentangled latent representation by adding the similarity constraint on the generator
*Latent representation based GANs*	DEGAN [116]	Utilize the pretrained decoder and encoder structure from VAE to transform random Gaussian noise to distribution that contains intrinsic information of the real images
	VAEGAN [115]	Combine VAE and GAN
	AAE [113]	Impose discriminator on the latent space of the autoencoder architecture
	VEEGAN [117]	Add reconstruction network that reverse the action of generator network to address the problem of mode collapse
	BiGAN [114]	Attach encoder component to learn inverse mapping of data space to latent space
*Stack of GANs*	LAPGAN [132]	Introduce Laplacian pyramid framework for an image detail enhancement
	MADGAN [135]	Use multiple generators to discover diverse modes of the data distribution
	D2GAN [134]	Employ two discriminators to address the problem of mode collapse
	CycleGAN [137]	Use two generators and two discriminators to accomplish unpaired image to image translation task
	CoGAN [136]	Use two GANs to learn a joint distribution from two-domain images
*Other variants*	SAGAN [141]	Incorporate self-attention mechanism to model long range dependencies
	GRAN [133]	Recurrent generative model trained using adversarial process
	SRGAN [139]	Use very deep convolutional layers with residual blocks for image super resolution

### Objective variants

The main objective of GAN is to approximate the real data distribution. Hence, minimizing distance between the real data distribution $\left( {p_{r} } \right)$ and the GAN generated data distribution $(p_{g} )$ is a vital part of training GAN. As stated in “Deep Generative image models” section, standard GAN [67] uses Jensen Shannon divergence to measure similarity between real and generated data distributions $D_{JS} (p_{r} ||p_{g} )$. However, JS divergence fails to measure distance between two distributions with negligible or no overlap. To improve performance and achieve stable training of GAN, several distances or divergence measures have been proposed instead of JS divergence.

*WGAN *Wasserstein Generative Adversarial Network (WGAN) [123] replaces JSD from the standard GAN with the Earth mover Distance (EMD). EMD also known as Wasserstein Distance (WD) can be interpreted informally as minimum amount of work to move earth (quantity of mass) from the shape of one distribution p(x) to that of another distribution q(x) so as to match shape of both the distributions. WD is smooth and can provide meaningful distance measure between distributions with negligible or no overlap. WGAN imposes an additional Lipchitz constraint to use WD as the loss in the discriminator, where it deploys weight clipping to enforce weights of the discriminator to satisfy Lipchitz constraint after each training batch.

*WGAN-GP *Weight clipping in the discriminator of a WGAN greatly diminishes its capacity to learn and often fails to converge. WGAN-GP [124] is an extension of WGAN that replaces weight clipping with gradient penalty to enforce discriminator to satisfy Lipchitz constraint. Furthermore, Petzka et al. [125] proposed a new regularization method, also known as WGAN-LP, that enforces the Lipschitz constraint.

*LSGAN *Least squares Generative Adversarial Network (LSGAN) [126] deploys least square loss instead of the cross entropy loss in discriminator of the standard GAN to overcome the problem of Vanishing gradient as well as improving quality of generated image.

*EBGAN* Energy Based GAN (EBGAN) [127] uses auto-encoder architecture to construct the discriminator as an energy function instead of a classifier. The Energy of EBGAN is the mean squared reconstruction error of an autoencoder, providing lower energy to the real images and high energy to generated images. EBGAN exhibits faster and more stable behavior than standard GAN during training.

Same as EBGAN, Boundary Equilibrium GAN (BEGAN) [128], Margin adaptation GAN [129] and dual agent GAN [130] also deploy an auto-encoder architecture as the discriminator. The discriminator loss of BEGAN uses Wasserstein distance to match the distributions of the reconstruction losses of real images with the generated images.

There are also several other objective functions based on Cramer distance [144], Mean/covariance Minimization [145], Maximum mean discrepancy [146], Chi-square [147] have been proposed to improve performance and achieve stable training of GAN.

### Training tricks

While research on various GANs architectures and objective functions continue to improve the stability of training, there are several training tricks proposed in the literature intended to achieve excellent training performance. Radford et al. [112] showed using leaky rectified activation functions in both generator and discriminator layers gave higher performance over using other activation functions. Salimans et al. [148] proposed several heuristic approaches which can improve the performance, and training stability of GANs. First, feature matching, changes the objective of the generator to minimize the statistical difference between features of the generated and real images. In this way, the discriminator is trained to learn important features of the real data. Second, minibatch discrimination, where the discriminator process batch of samples, rather than in isolation that helps prevent mode collapse, as the discriminator can identify if the generator continues to generate sample with little variety. Third, historical averaging, that takes the running average of parameters in the past and penalizes if there is a large difference between parameters, which can help the model to converge to an equilibrium. Finally, one-sided label smoothing provides smoothed labels to the discriminator instead of 0 or 1, which can smooth the classification boundary of the discriminator.

Sønderby et al. [149] proposed the idea of crippling the discriminator by introducing noise to the samples rather than labels, which prevents the discriminator from overfitting. Heusel et al. [150] used a separate learning rate for generator and discriminator, and trained GANs by a Two Time-Scale Update Rule (TTUR) to ensure that model converge to a stationary local Nash equilibrium. To stabilize the training of the discriminator, Miyato et al. [151] proposed normalization technique called spectral normalization.

## Taxonomy of class imbalance in visual recognition tasks

This section describes different GANs applied to imbalance problems in various visual recognition tasks. We group the imbalance problems in a taxonomy with three main types: 1. Image level imbalances in classification 2. object level imbalances in object detection and 3. pixel level imbalances in segmentation tasks. Understanding this taxonomy of imbalances will provide a valuable framework for further research into synthetic image generation using GAN.

### Class imbalances in classification

Image classification is the task of classifying an input image according to a set of possible classes. Classification can be broken down into two separate problems: binary classification and multi-class classification. Binary classification involves assigning an input image into one of two classes, whereas in multi-class classification two or several classes are involved. A classic example of a binary image classification problem is the identification of cats or dogs in each input image. Image dataset with high imbalance [152], which includes inter-class imbalance and intra-classes imbalance, results in poor classification performance.

#### Inter class imbalance

Inter-class imbalance refers to a binary image classification problem where a minority class contains a smaller number of instances when compared to instances belonging to the majority class. Inter class imbalance in a dataset is described in terms of the imbalance ratio. The ratio between the numbers of instances of the majority class and those of the minority class is called the imbalance ratio (IR). For example, binary class imbalance with imbalance ratio of 1:1000 means that for every one-instance in a minority class, there are 1000 instances in the majority class. Datasets with a high imbalance ratio are harmful because they bias the classifier towards majority class predictions.

**Fig. 11:**
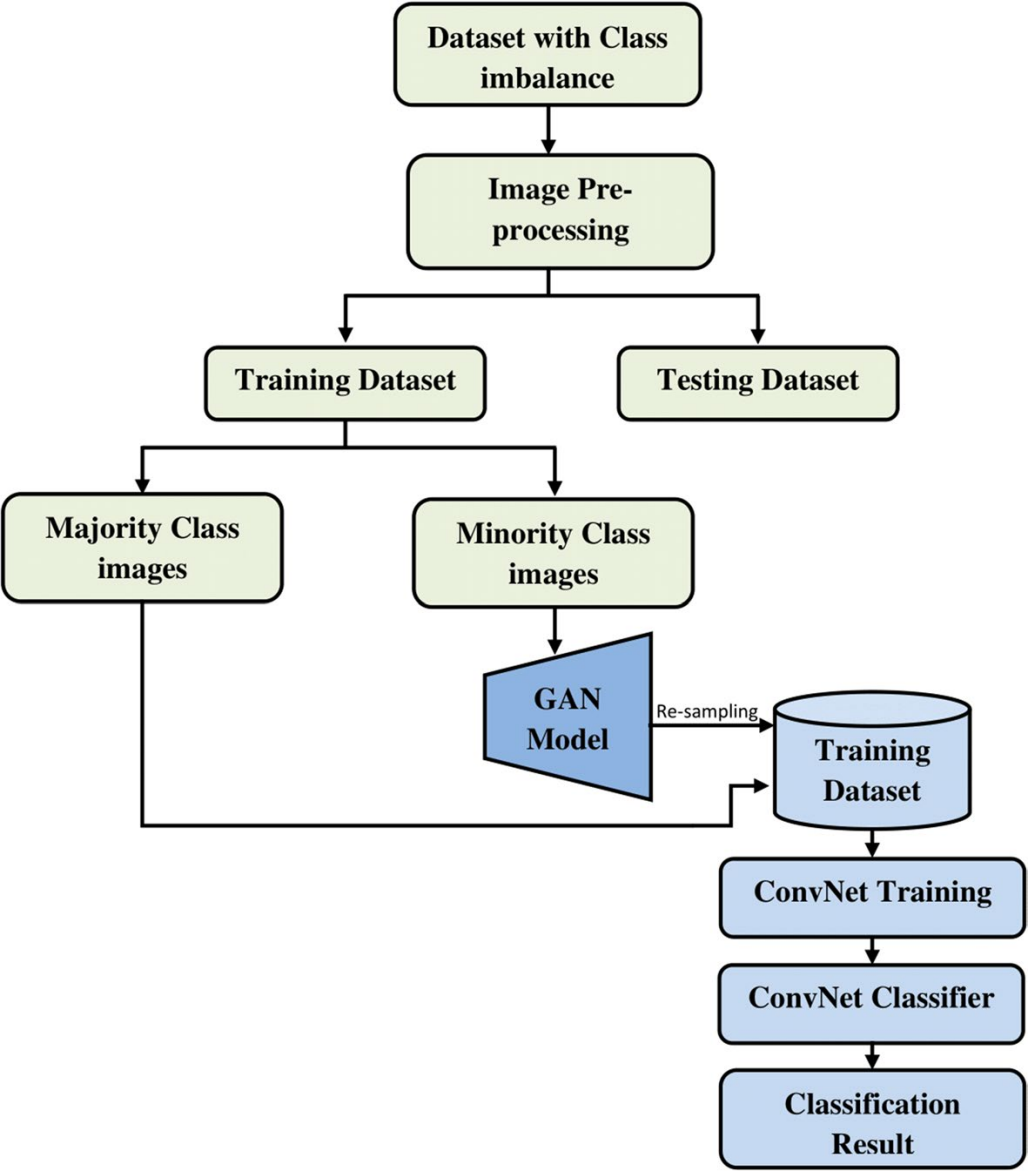
flowchart of GAN-based oversampling technique

Synthetic images generated using GAN can be used as an intelligent oversampling technique to solve class imbalance problems. The general flowchart of GAN-based oversampling technique is depicted in Fig. 11. This GAN-based oversampling technique not only increases the representation of the minority class, but it may also help to prevent over fitting.

Shoohi et al. [153] have used DCGAN to restore balance in the distributions of imbalanced malaria dataset. Generated synthetic images from DCGAN are used to achieve 100% balance ratio by oversampling minority class and thus reduce the false positive rate of classification. Their original dataset contains 18,258 cell images, (13,779 parasitized cells, 4,479 uninfected cells). After using an imbalanced dataset to achieve 50% accuracy, they observed an increase to 94.5% accuracy once they added the DCGAN-generated samples.

Niu et al. [154] introduced surface defect-generation adversarial network (SDGAN), using D2 adversarial loss and cycle consistency loss for industrial defect image generation. SDGAN is trained to generate defective images from defect-free images. D2 adversarial loss enables the SDGAN to generate defective images of high image quality and diversity, while cycle consistency loss helps to translate defective images from defect-free images. Surface defect classifier trained on the images synthesized by the SDGAN achieved 0.74% error rate and, also proved to be robust to uneven and poor lighting conditions.

Mariani et al. [155] argued that the few examples in minority class may not be sufficient to train GANs, so they introduced a new architecture called Balancing GAN (BAGAN). BAGAN utilizes all available images of minority and majority classes, and then tries to achieve class balance by implementing class conditioning in the latent space. Learning useful features from majority classes can help the generative model to generate images for minority classes. An autoencoder is employed to learn an exact class-conditioning in the latent space.

Most of the work done in utilizing GANs based synthetic images for class imbalance and comparing the resulting classification performance have been performed in medical image datasets [152, 156–158], and [159]. In the study of Wu et al. [156], class conditional GAN with mask infilling (ciGAN) is trained to generate examples of mammogram lesions for addressing class imbalance in mammogram classification. Instead of generating malignant images from scratch, ciGAN simulates lesions on non-malignant images. For every non-malignant image, ciGAN generates a malignant lesion onto it using a mask from another malignant lesion. On the DDSM (Digital Database for Screening Mammography) Dataset [152], synthetic images generated using ciGAN improves classification performance by 0.014 AUC over baseline model and 0.009 AUC compared to standard augmentation techniques alone.

The vast majority of studies in bio-medical domain used cycle-GAN [138] to generate synthetic medical images. Muramatsu et al. [157] tested the use of a cycle-GAN to synthesis mammogram lesion images from different organs in mammogram classification. They translated CT images with lung nodules to mammogram lesion images using cycle-GAN and found classification accuracy improved from 65.7% to 67.1% with generated images.

For breast cancer detection, Guan and Loew [158] compared the usefulness of DCGAN-generated mammograms and traditional image augmentation method in a mammogram classification task. On the DDSM Dataset [152], the GAN based oversampling method performed about 3.6% better accuracy than traditional image augmentation techniques.

Most recently, Waheed et al. [159] proposed a variant of ACGAN, called CovidGAN for the generation of synthetic Chest X-Ray (CXR) images to restore balance in the imbalanced dataset. Their dataset contains 721 images of Normal CXR and 403 images of Covid-CXR collected from three publicly accessible databases: (1) COVID-19 Chest X-ray Dataset Initiative [160], (2) IEEE Covid Chest X-ray dataset [161] and (3) COVID-19 Radiography Database [162]. The generator network in the CovidGAN is stacked on top of the discriminator. At the beginning of the training process, the layers of the discriminator are freezed and thus, only the generator network gets trained via the discriminator. However, the author offers no explanation for the significance of stacking. They observed improved classification accuracy from 85 to 95% when the classifier is trained on combination of original and synthetic images.

The effectiveness of using synthetic images to balance the class distribution is fairly a recent idea that has not been widely tested and understood. At low resolution image datasets, adding synthetic images with original images have shown to improve performance of the classifiers, but at the higher resolution image datasets these synthetic images become obvious to distinguish from the real one. This is due to the fact that the higher resolution images allow for finer textures and details, and hence will need more cautious modifications by GAN so as not to distort the natural patterns occurring in the high-resolution image dataset. Improving the resolution of GAN samples and testing their effectiveness is an interesting area of future work.

#### Intra class imbalance

Another type of imbalance that deteriorates performance of the classification problem is the intra-class imbalances. The techniques used for inter-class imbalance can be extended to intra-class imbalance if the datasets have detailed labels. However, in real world datasets, data acquisition with a detailed label is rare because acquiring detailed dataset is costly, and sometimes even not feasible [163]. In many cases, collecting images is tiresome, like 1. capturing images of the same person with glasses and without them, 2. Images of the same person face with varying poses, facial attributes, etc. In some cases, such as the gender swapping, it is not feasible to collect images of the same person as both male and female. Therefore, those techniques for inter-class imbalance are hard to solve intra-class imbalance.

**Fig. 12:**
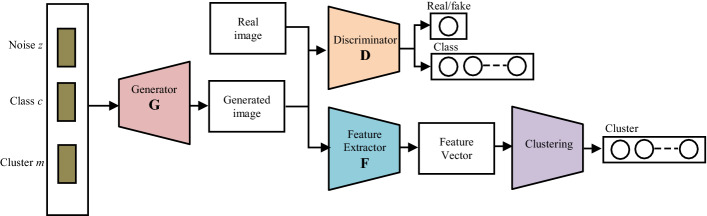
Architecture diagram of clustering based GAN for solving intra-class imbalance presented by Hase et al. [163]

Hase et al. [163] presented an interesting idea to combine clustering technique with GANs designed for solving intra class imbalance. The proposed architecture consists of the generator $G$, the discriminator $D$, and the pre-trained feature extractor $F$ (Fig. 12). The key idea is to generate clusters of images in each class in the feature space, and synthesize images conditioned on class and cluster while estimating the clusters of generated images. The generator $G$ is trained to generate an equal number of images for each class and cluster, so that the distribution of both inter and intra class become uniform.

Utilizing clustering techniques in the feature space to divide the images into groups for an automatic pattern recognition in the dataset is a promising area for future work. Additionally, it will be interesting to see how the performance of GAN changes with different types of clustering methods such as Hierarchical clustering, Fuzzy clustering, Density-based clustering, etc.

A semantically decomposed GAN (SD-GAN) proposed by Donahueet al. [164] adopts Siamese networks that learn to generate images across both inter and intra class variations. Both GANs and Siamese networks have two networks. But unlike GANs, where the two networks compete with each other, the two networks in Siamese networks are similar and working one beside the other. They learn to compare output of the two networks on two different inputs and measure their similarity. For example, Siamese networks can measure the probability that two signatures are made by the same person. A combination of GAN and Siamese networks in SD-GAN can learn to synthesize photorealistic variations (such as, viewpoints, light conditions, scale, backgrounds, and more) of an original input image.

Many studies have reported the problem of an intra-class imbalance owing to age, gender, race and pose attribute variations in face recognition tasks [165–168]. Several variants of GAN have been proposed to address this issue, some focusing on modifying one or more facial attributes, others on generating high quality face images with distinctive pose variations.

##### Facial attribute editing

**Fig. 13:**
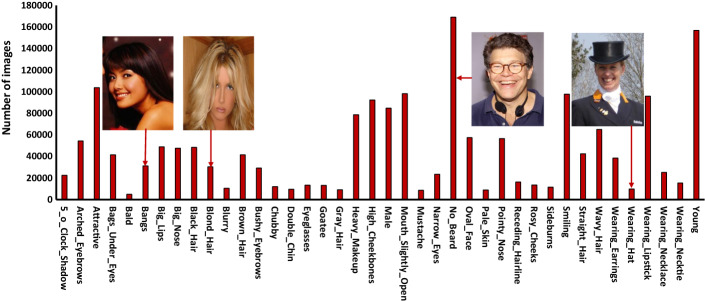
Imbalanced distribution of 40 binary face attributes (positive and negative) on CelebA dataset [169]

Human face attributes are highly imbalanced in nature. Attributes can be combined to generate descriptions at multiple levels. For instance, one can describe “white-female” at the category level, or “white-female blond-hair black-eyes wearing necklace” at the attribute level. Attribute level imbalances are inevitable in facial recognition datasets (Fig. 13). As an example, Bald persons with a mustache wearing neckties are 14 to 45 times less likely to occur in the CelebA dataset [169].

Face attribute editing aims to edit the face image by modifying single or multiple attributes while preserving other details. It is challenging because some of the face attributes are locally distributed, such as ‘bangs’, ‘wavy hair’, and ‘mustache’, but some are globally attributed such as ‘chubby’, ‘smiling’ and ‘attractive’. Several GANs based methods have been proposed to achieve face attribute editing tasks.

Anders et al. [115] proposed a model that combines VAE and GAN together and learns to map the facial images into latent representation. The derived latent representations are then used to find the attribute manipulating direction. For a given facial attribute (e.g., blond hair), the training dataset can be separated into two groups that images with or without blond hair, then the manipulation direction can be computed as the difference between the mean latent representation of two groups. However, such latent representation contains highly correlated attributes, that results in unexpected changes of other attributes, e.g., adding mustache always makes a female become a male as mustache objects are always associated with male in the training set.

**Fig. 14:**
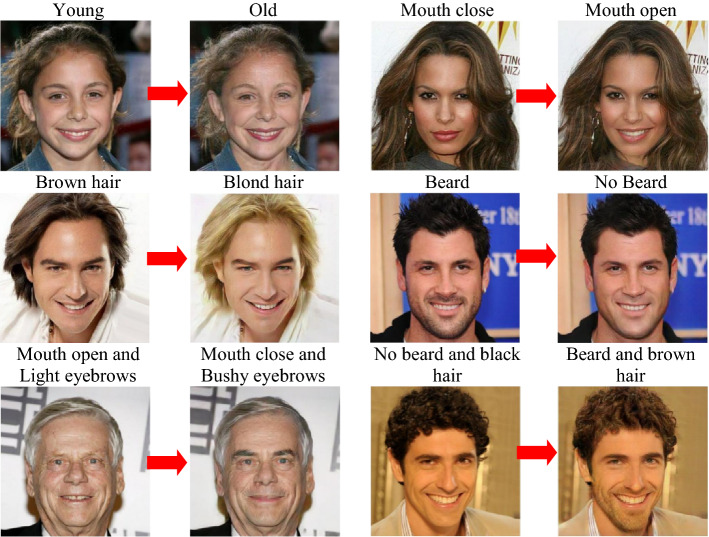
Face attribute editing examples created by AttGAN [64]

He et al. [64] showed how single or multiple facial attributes of a face image can be manipulated by using encoder-decoder architecture. i.e., to generate and modify a face image with the required attributes, while preserving realism of the image (Fig. 14). They have introduced encoder-decoder architecture in GAN to handle this task. Encoder in the encoder-decoder architecture maps a facial image onto a latent representation and facial attribute editing is accomplished by decoding the latent representation conditioned on the expected attributes. The authors applied an attribute classification constraint to guarantee that the attributes are correctly edited. Meanwhile, reconstruction learning is employed to ensure the attributes excluding details are well preserved.

**Fig. 15:**
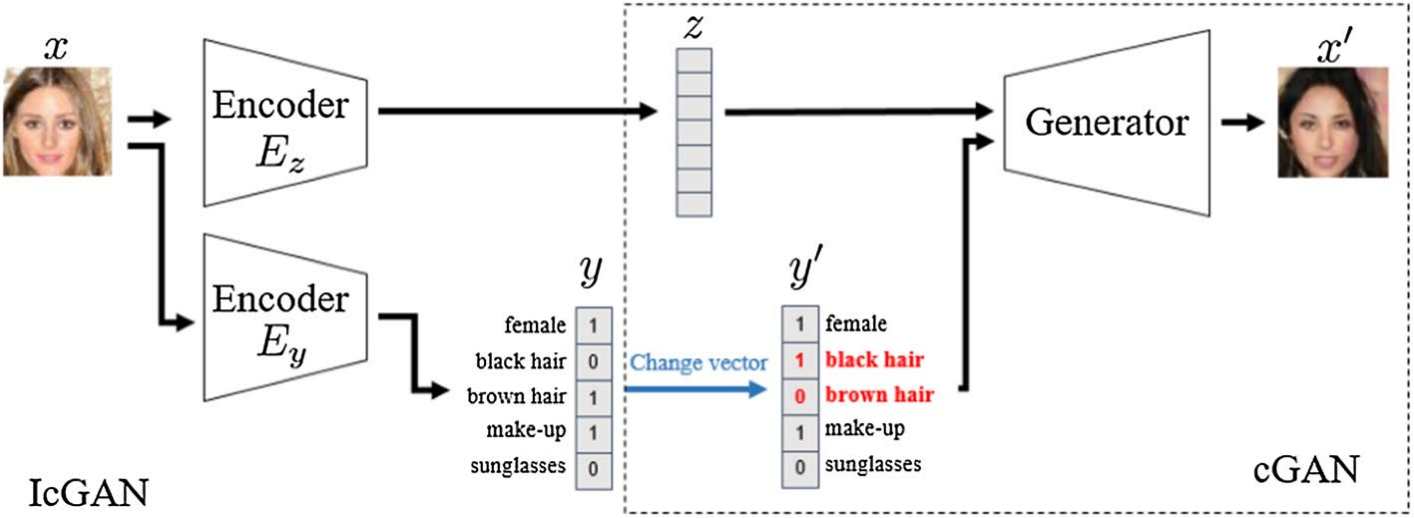
illustration of invertible conditional GAN presented by Perarnau et al. [65]

Perarnau et al. [65] proposed an invertible conditional GAN (IcGAN) that is equipped with two encoders to inversely map from input facial images into conditional vector $y$ and latent vector $z$, which, as a result can be manipulated to generate a new face image with desired attributes. IcGAN is a multi-stage training algorithm that first trains a cGAN [118] to map from conditional vector $y$ and latent vector $z$ to real images, and in a second step learns its inverse mapping from generated images to conditional vector $y$ and latent vector $z$ in a supervised manner (Fig. 15). In this way, by changing the conditional vector $y$, IcGAN allows to control attribute relevant features (e.g. hair color) while latent vector $z$ allows to modify attribute irrelevant features (e.g. pose, background).

Tao et al. [66] argued that the facial attribute editing is an image-to-image translation problem, which aims to transfer facial images from the source domain to the target domain. Their proposed model contains three major parts: an encoder, a decoder, and a residual attributes extractor. The encoder and decoder together constitute a generator, whose main aim is to generate a facial image with desired attributes. The encoder maps the facial images into latent representation and the decoder reconstructs (generates) the image from this representation along with attribute vectors. The main purpose of residual attributes extractor is to learn the gap between the original input and the desired output in the feature space and back propagate error signal to supervise the generation process.

Zhangi et al. [170] have used the design principle of Adversarially Regularized U-net (ARU-net), instead of conventional encoder and decoder architecture to learn facial attribute editing and generation tasks together during training. The symmetric skip connection technique is used to pass on the details from encoder to decoder, which preserves the attribute irrelevant features. In this architecture, the ARU-net is integrated with GANs that results in ARU-GAN to perform facial attribute editing. The ARU-GAN consists of four major components: the ARU-net for preserving attribute irrelevant features, the adversarial network to constrain the latent representation, the discriminator to distinguish between real and fake image, and the attribute classifier to ensure the desired attributes are edited.

Zhang et al. [171] introduced a spatial attention mechanism into GANs for only modifying attribute relevant parts and keeping attribute irrelevant parts unchanged. SaGAN [141] is used to locate and manipulate attribute-relevant part more precisely. The generator of the proposed architecture consists of an attribute manipulation network (AMN) and a spatial attention network (SAN). Given a face image, SAN learns to localize the attribute-specific region and then AMN edit the face image with the desired attributes in the specific region located by SAN.

The major downside with the current approaches is that the input to GAN should be frontal face images. It will be interesting to explore a new architecture that can be trained to modify the attributes of side-view or any arbitrary views.

##### Person re-identification

**Fig. 16:**
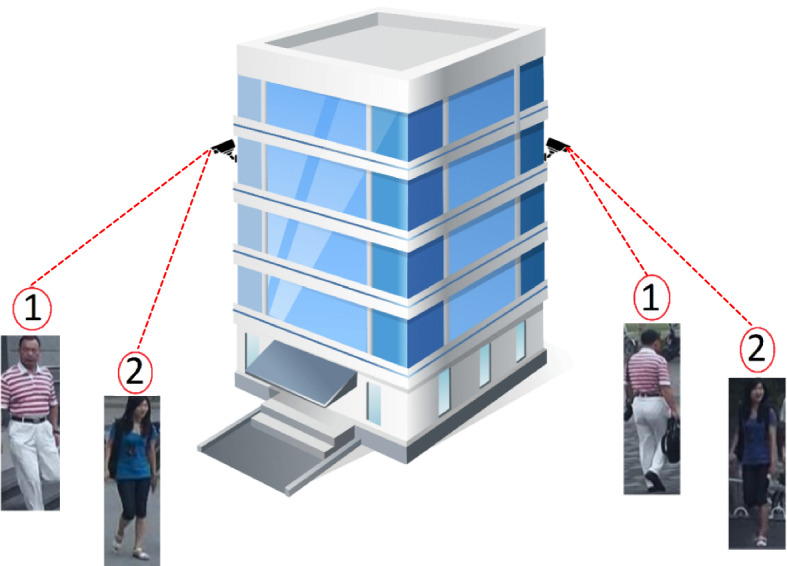
Example of Person reidentification task. Person reidentification is a key element in video surveillance that deals with matching images of same person over many non-overlapping camera views

Person re-identification [172] is another challenging task worth mentioning, which are adversely affected due to significant intra class imbalance. Intra class variations caused by rotation (varying poses) are often larger than the inter-person dissimilarities used to differentiate the face images [173]. Recent face-recognition surveys [174, 175] identified pose variation as one of the prominent unresolved issues in face-recognition task. For instance, in order to maintain the highest standard of security, a smart video system needs to be able to detect a person invariant to pose (Fig. 16).

**Fig. 17:**
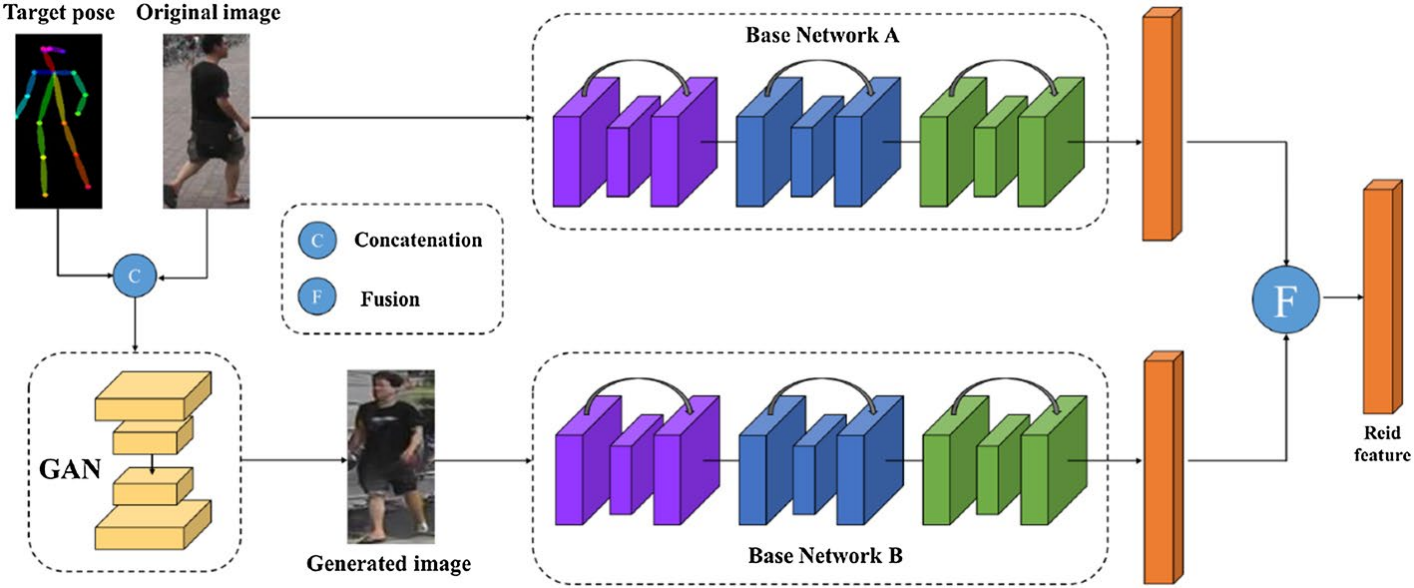
Architecture diagram of pose-normalized GAN presented by Qian et al. [176]

Qian et al. [176] introduced a pose-normalized GAN model (PN-GAN) for alleviating the effects of pose variation. Given any pedestrian image and a desirable pose as input, the model utilized a desirable pose to produce a synthetic image of the same identity with the original pose replaced with the desirable pose (Fig. 17). After this, the authors trained the re-identification model with the original images and generated pose-normalized images to extract two sets of features. Finally, they fused the two types of features as the final feature. As a result, the features extracted from the synthesized images improved the generalization ability of the re-identification model.

To address person re-identification challenges in complex scenarios, Wei et al. [177] proposed a model called Person Transfer Generative Adversarial Network (PTGAN) for implausible person image style transfer from source domain to target domain, across datasets with different styles, such as backgrounds, poses, seasons, lightings, etc. The domain transfer procedure in PTGAN is inspired by CycleGAN [138]. Different from Cycle-GAN [138], PTGAN incorporates additional constraints on the person foregrounds to make sure the stability of their identities during transfer. Compared with Cycle-GAN, PTGAN generates high resolution person images, where person identities are unchanged, and the styles are transformed.

Being a cross-camera tracking and human retrieval task, person re-identification often suffers from image style variations resulting from different cameras. Therefore, Zhong et al. [178] designed a camera style adaption model for adjusting ConvNet training. They have used CycleGAN [138] for transferring images from one camera to the style of another camera. Given that both original and style transferred images, identification discriminative embedding (IDE) is used to train the ConvNet model. Particularly, authors have used ResNet-50 pre-trained on ImageNet dataset as backbone and follow the fine-tuning strategy.

Pedestrian images suffer from information loss when transferring from one camera to the style of another camera. Deng et al. [179] presented a model, named similarity preserving cycle consistent generative adversarial network (SPGAN), which is composed of a CycleGAN and a Siamese network (SiaNet). CycleGAN learns to translate pedestrian images from one domain to another domain, and the contrastive loss induced by the SiaNet pulls close a translated image and its counterpart in the source domain, and moves away the translated image and any image in the target domain.

Ge et al. [180] presented a Feature Distilling Generative Adversarial Network (FD-GAN) that aims at learning identity related and pose-unrelated person representations. The proposed model adopts a Siamese structure with multiple novel discriminators on human poses (pose discriminator) and identities (identity discriminator). The idea behind FD-GAN is to learn pose-unrelated and identity-related features of pedestrian image, then it can be used to generate the same pedestrian image but with different target poses.

Although GAN-based methods described above have achieved excellent performance in image-based person re-identification, it still needs considerable effort to tackle the video-based identification datasets. Future work seeks to expand to use GAN for generating a sequence of images for the video-based identification datasets.

##### Vehicle re-identification

**Fig. 18:**
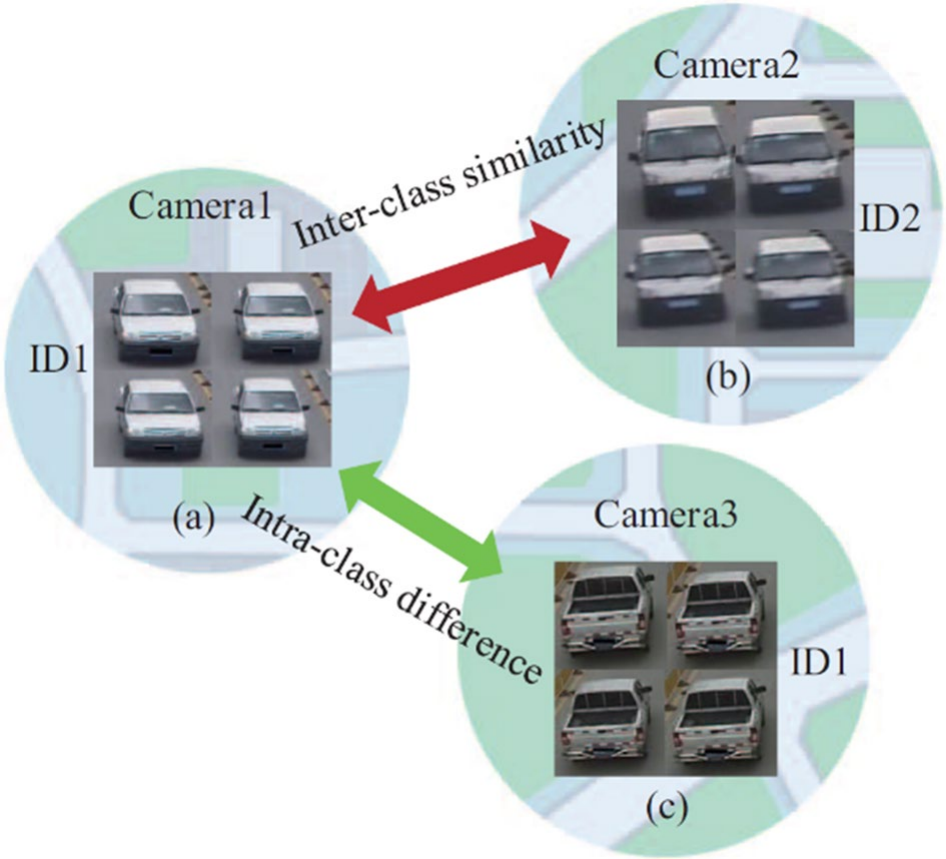
illustration of challenges in vehicle Re-identification provided by Zheng et al. [181]

Vehicle Re-identification task is even more challenging as it suffers from large intra-class differences caused by viewpoint and illuminations variations, and inter-class similarity primarily for different identities with the similar look (Fig. 18).

Zhou et al. [182] proposed a model called Cross view GAN to generate images in different viewpoints of the same vehicle. Cross view GAN composed of classification, generator, and discriminator network. First, classification network is trained to learn vehicle intrinsic features such as model, color, and type information. In addition to intrinsic features, it also learns viewpoint features. Then the generative network is conditioned on the average feature of the expected viewpoint and vehicle’s intrinsic features to infer images of the same vehicle in other viewpoints. The discriminator network learns to distinguish real images from the generated images, while ensuring images are generated with correct attributes.

Wu et al. [183] improved the discriminative power of the ResNet-50 model for the Vehicle re-ID task by simultaneously training with initial labeled images and DCGAN generated unlabeled images. They further explore the effectiveness of using DCGAN generated images on a wide range of vehicle re-ID datasets and show improved performance of vehicle re-identification.

##### Fine-grained image classification

**Fig. 19:**
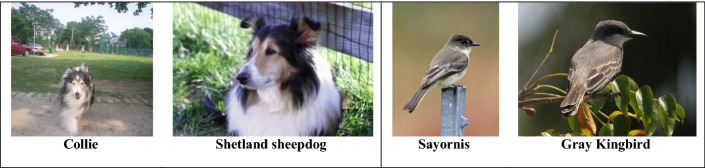
Sample images from the Stanford Dogs dataset [186] and the Caltech-UCSD Birds dataset [187], which exhibits minor inter-class variations and major intra-class variations

The fine-grained image classification is also attributed to major variations in the intra-class and minor inter class variations [184]. It is a difficult task for two reasons. First, the training samples of each class are inadequate. Second, the differences between different classes of images are quite small [185]. As an example, it is very difficult to identify the images of Shetland Sheepdog from that of Collie dog. Similarly, the images of Sayornis and Gray Kingbird are quite difficult to distinguish (Fig. 19).

Fu et al. [184] developed a model called Fine grained conditional GAN (F-CGAN) to solve fine grained class dependent image synthesis problems. F-CGAN consists of three main components: 1. a 2-stage GAN, 2. a fine-grained feature preserver and 3. a multi-task classification model. The 2-stage GAN generates high resolution images, the fine-grained feature preserver targets to capture fine grained details and the multi-task classification model utilizes generated image data to improve fine grained classification accuracy.

**Fig. 20:**
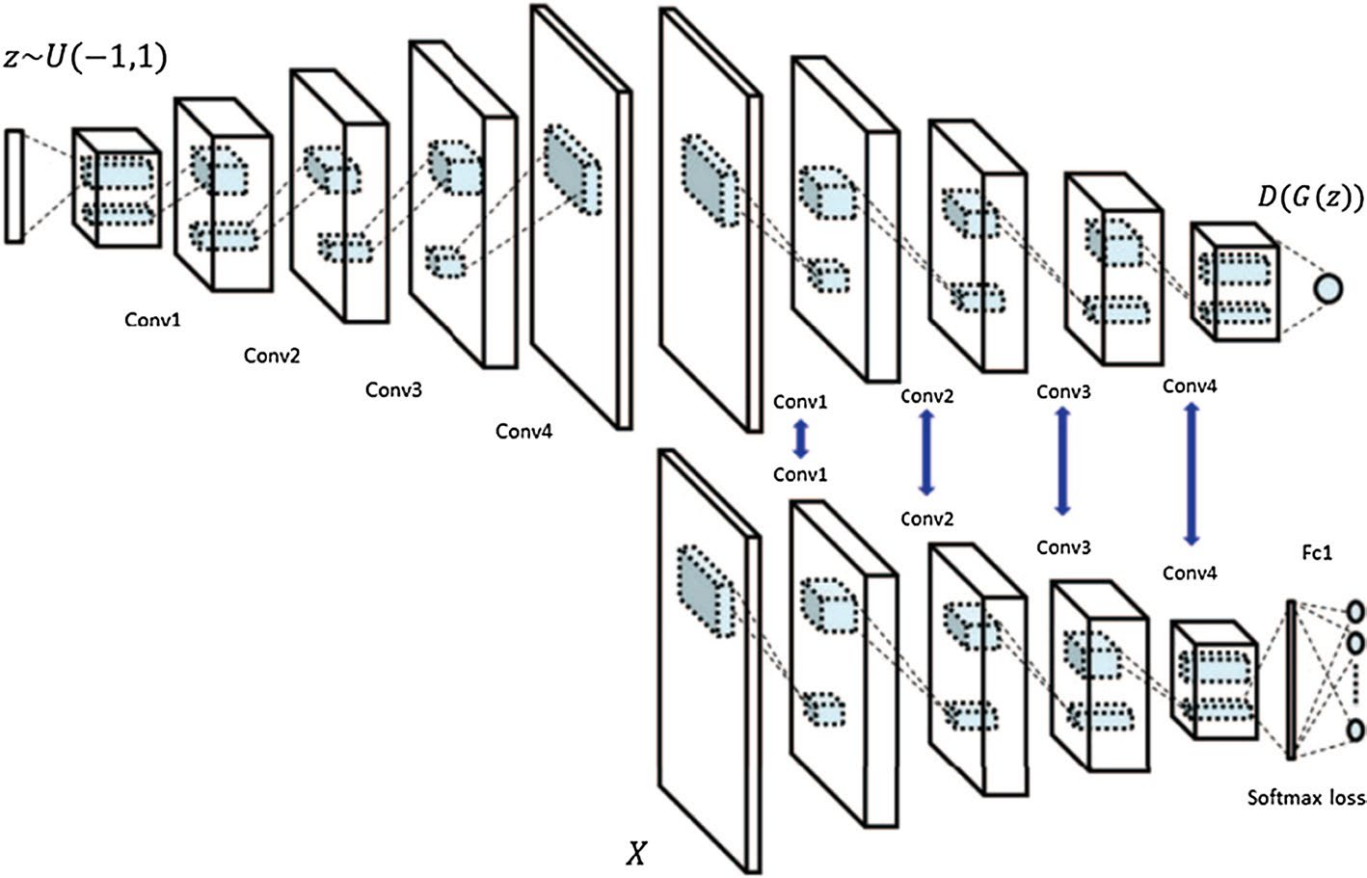
Complete fine-grained Plankton classifier architecture used by Wang et al. [188]

Wang et al. [188] find that the discriminator in GANs learns a hierarchical identification features of the fine-grained classes and discriminate pattern of the fine-grained training samples. They use the architecture pictured below to implement the fine-grained Plankton classification task (Fig. 20). The main idea is to train a fine-grained classifier that shares weights with discriminator of the DCGAN, which forces discriminator to concentrate on features of small classes. On WHOI-Plankton dataset [189], F1 score of the classifier improved by over 7%.

Typically, medical image datasets contain both general labels, e.g., “male”, “female” and disease specific detailed labels [190]. It is mentioned that the complexity and nature of data is hard to learn by using a single GAN. Hence, T. Koga et al. [190] connected two GANs in series, one for learning general features and other for detailed features. The first GAN generates diverse images, which takes a noise vector and general labels as inputs. The second GAN receives synthetic images generated by the first GAN, and disease specific detailed labels as inputs, and generates the final fine-grained medical images.

#### Multiclass imbalance

In many real world problems such as emotion classification [191], plant disease classification [192], medical image classification [193], industrial defect classification [194] etc., it is more likely that more than one class exists and needs to be recognized. Multiclass classification has been shown to suffer more learning difficulties than binary class classification, because multiclass classification increases the data complexity and intensifies the imbalanced distribution [195]. Three types of imbalance could occur to the multiclass datasets: few minority-many majority classes, many minority-few majority classes, and many minority-many majority classes. Shuo Wang et al. [196] studied the impact of all different types of multiclass imbalances and showed that they negatively affect minority class and overall performance.

**Fig. 21:**
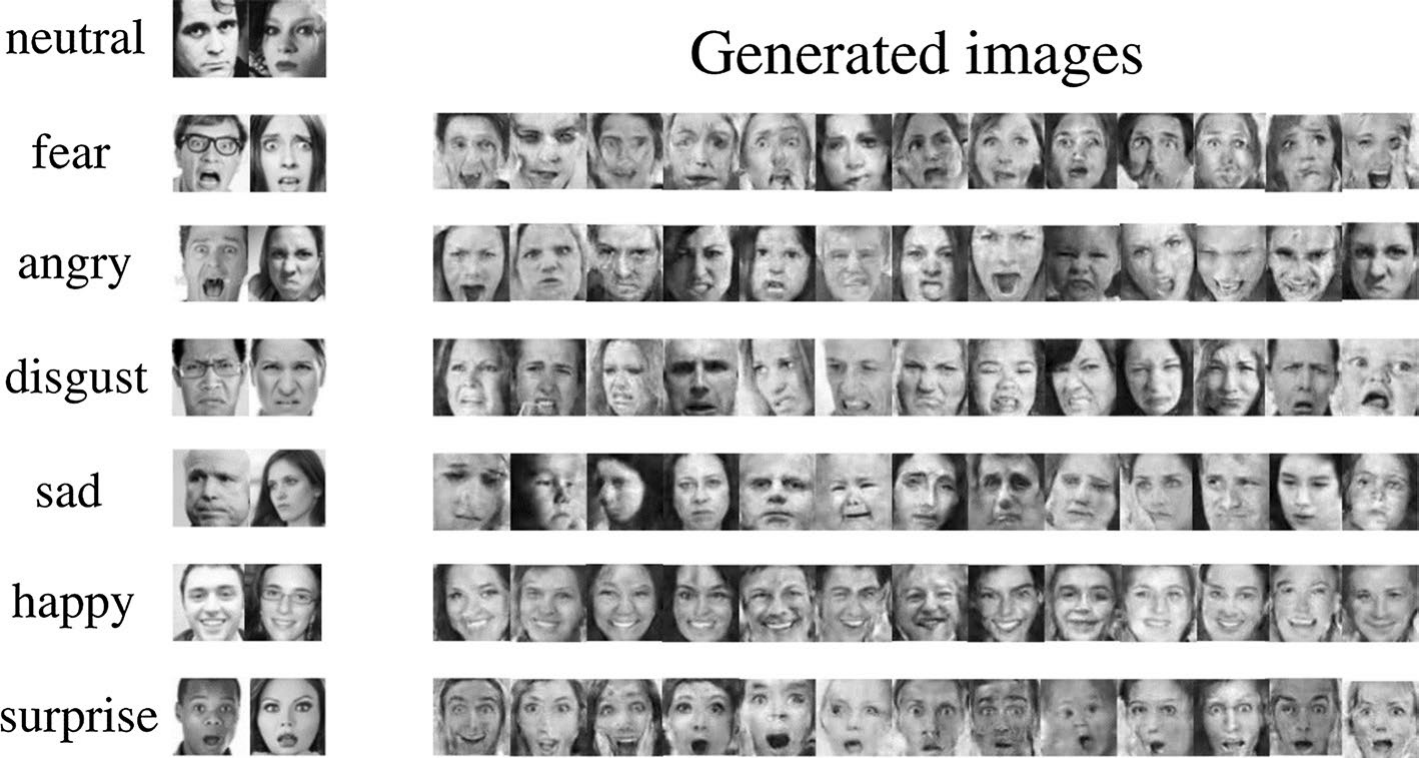
On emotion classification task [197], the images on the left are original data and the rest are images generated by cycle-GAN

An example of few minority-many majority class imbalance is an emotion classification, as some classes of emotions like disgust are relatively uncommon compared to common emotions like happy or sad. Zhu et al. [197] employed cycle-GAN which can synthesize uncommon emotion classes like disgusted from the frequent classes (Fig. 21). In addition to adversarial and cycle consistency loss, they use least square loss from LSGAN to avoid vanishing gradient problems. Employing cycle-GAN based data minority class data augmentation achieved 5–10% increase in the overall accuracy. They also found that enlarging minority classes also increases accuracy of other majority classes.

Weather Image classification is another example of few minority-many majority class imbalance, because some types of weather, like snow, is relatively rare compared to sunny, hazy and rainy days. Li et al. [198] used DCGAN to generate images of minority classes in training. They found that the GAN-based data augmentation technique led to margin clarity between classes and hence improvement in classification performance.

**Fig. 22:**
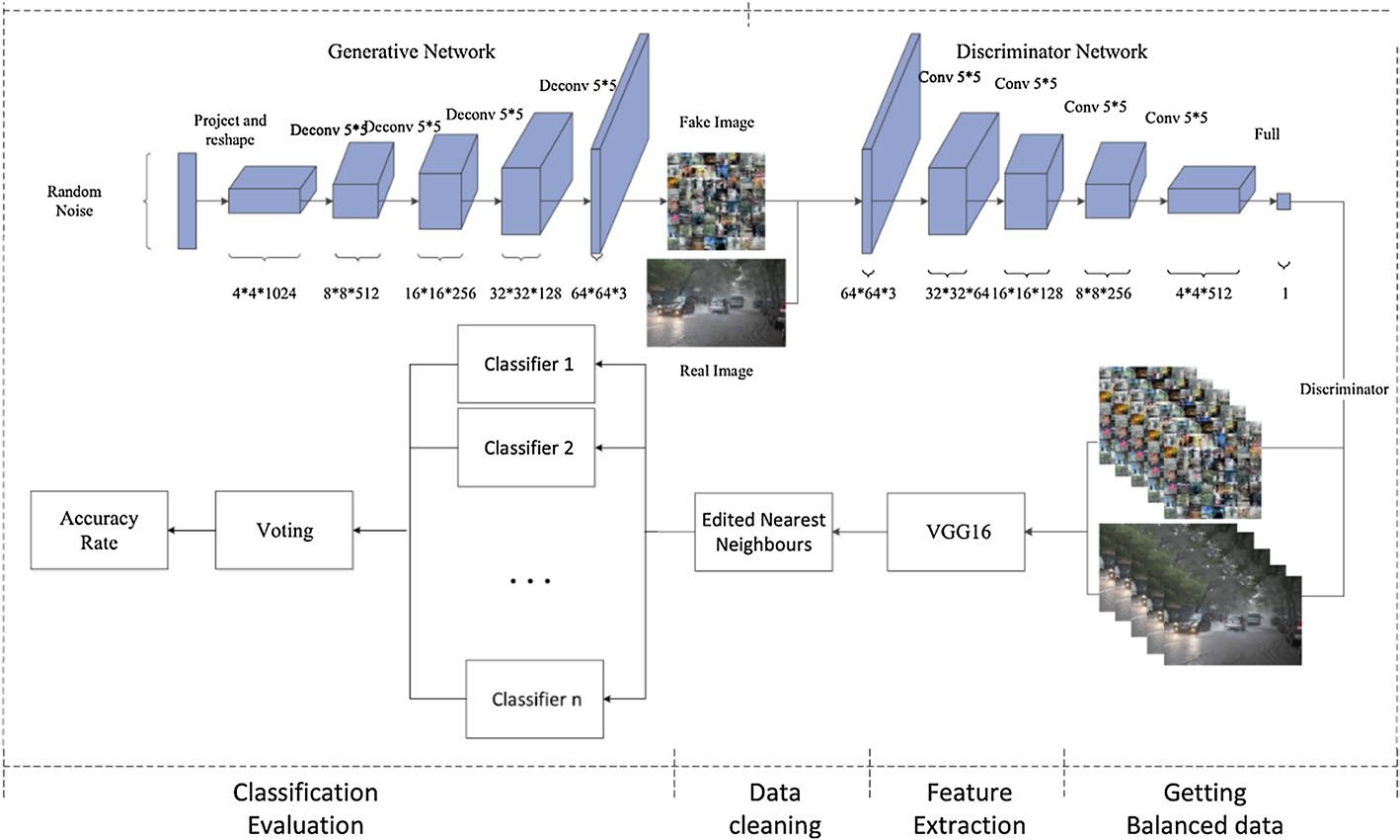
Illustration from Huang et al. [199] showing how the Ensemble learning is integrated with GAN Framework

Huang et al. [199] presented an interesting idea to combine ensemble learning with GANs designed to address the class imbalance problem in weather classification. The proposed method comprised of three ingredients as depicted in (Fig. 22): 1. DCGAN to generate synthetic images and balance the training dataset 2. Nearest neighbor method to remove any possible outlier images generated by DCGAN 3. An ensemble learning method to combine the classification results of the multiple classifiers so as to achieve better results.

**Fig. 23:**
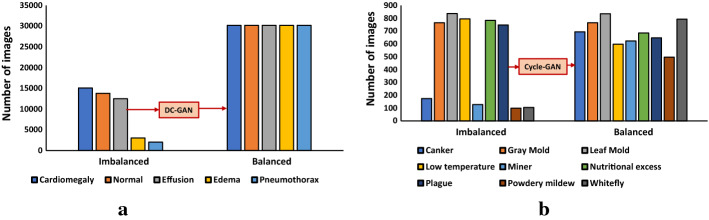
The distributions of (**a**) Chest X-ray image dataset [193] and (**b**) tomato plant disease dataset [192], before (left) and after class balancing using GANs (right)

The use of DCGAN was tested by Salehinejad et al. [193] in the task of chest pathology classification. Using chest X-ray images, they build a deep ConvNet classifier to classify 5 different anemic classes. Their dataset is highly imbalanced, contains three majority and two minority classes (Fig. 23a). The synthetic images generated using DCGAN were used to balance and augment the original imbalanced dataset. They demonstrated that a combination of the original imbalanced dataset and generated images improves the accuracy of deep ConvNet classifier in comparison to the same classifier trained with original imbalanced dataset alone. On chest X-ray dataset [193], a mean classification accuracy improved from 70.87 to 92.10%.

Frid-Adar et al. [200] also showed that generating synthetic liver lesion images using DCGAN can improve classification results. They combined standard augmentation techniques and DCGAN generated synthetic images to train a classifier. Their liver lesion dataset contains 182 computed tomography images (65 hemangiomas, 64 metastases and 53 cysts). By adding the synthetic images to standard data augmentation, their classification performance increased from 78.6% sensitivity and 88.4% specificity using standard augmentations to 85.7% sensitivity and 92.4% specificity using DCGAN-based synthetic images.

**Fig. 24:**
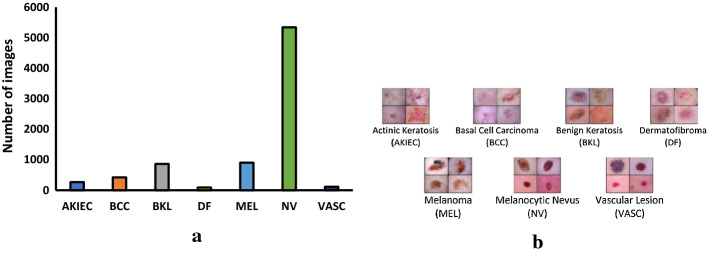
**a** Distribution of the seven skin lesion class labels of the ISIC 2018 dataset [202]. **b** Sample images from each class

Rashid et al. [201] tested the effectiveness of using GANs to generate skin lesion images. Using ISIC 2018 dataset [202], they built a CNN classifier to classify 7 different skin lesions as depicted in Fig. 24. These classes are highly imbalanced, and the GAN is used as a method of intelligent oversampling.

Nazki et al. [192] used Cycle-GAN to alleviate multiclass imbalance problem in tomato plant disease classification. Their tomato plant disease dataset contains 2789 images, highly suffered from class imbalance in 9 disease categories (Fig. 23b). Using Cycle-GAN, they translated images from the healthy tomato leaves to underrepresented diseased tomato leaves. This study demonstrated that the synthetic image generated by Cycle-GAN can be used as an augmented training set to improve the performance of classifier.

Bhatia et al. [203] sought out to compare synthetic images generated using WGAN-GP against the standard data augmentation in the context of multiclass image classification. They artificially introduced class imbalance in two balanced datasets of CIFAR-10 [87] and FMNIST [204], and studied the effects of multiclass imbalance on classification performance. On the CIFAR-10 [87] dataset, classification performance improved from 80.84% accuracy and 0.806 F1-score using standard data augmentation to 81.89% accuracy and 0.812 F1-score using WGAN-GP. On FMNIST [204] dataset, performance improved from 91.9% accuracy and 0.921 F1-score using augmentation to 92.8% accuracy and 0.923 F1-score using WGAN-GP.

**Fig. 25:**
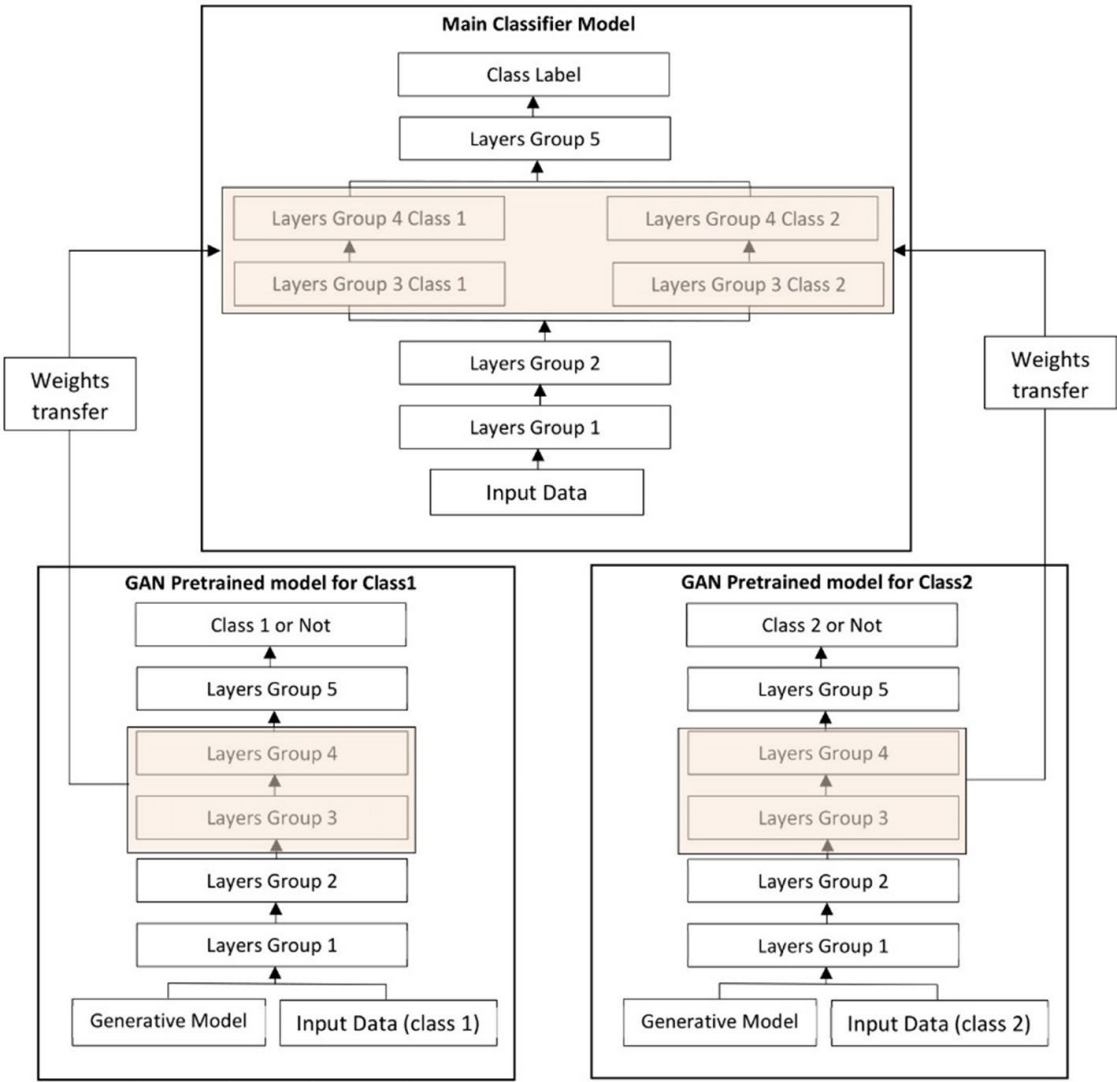
Illustration of the class expert generative adversarial network architecture [205]

An idea of GANs based transfer learning technique for multiclass imbalance problem is proposed by Fanny et al. [205]. Their architecture named class expert generative adversarial network (CE-GAN) makes use of multiple GANs models, a separate GANs for each class. Feature maps in the main classifier are arranged in parallel, with each feature maps pre-trained to identify the characteristics of a single class in the training data (Fig. 25). The weights of the pretrained feature maps are transferred from discriminators of the GANs to main classifier model for further training in a supervised mode.

The GAN-based synthetic images served as an intelligent oversampling technique and can address the problem of multi-class imbalance to a greater extent. However, synthetic images must be used with caution because if the quality of the synthesized images is not high, this would lead to additional noise to the original datasets.

### Object level imbalances in object detection

#### Object-scale imbalance

**Fig. 26:**
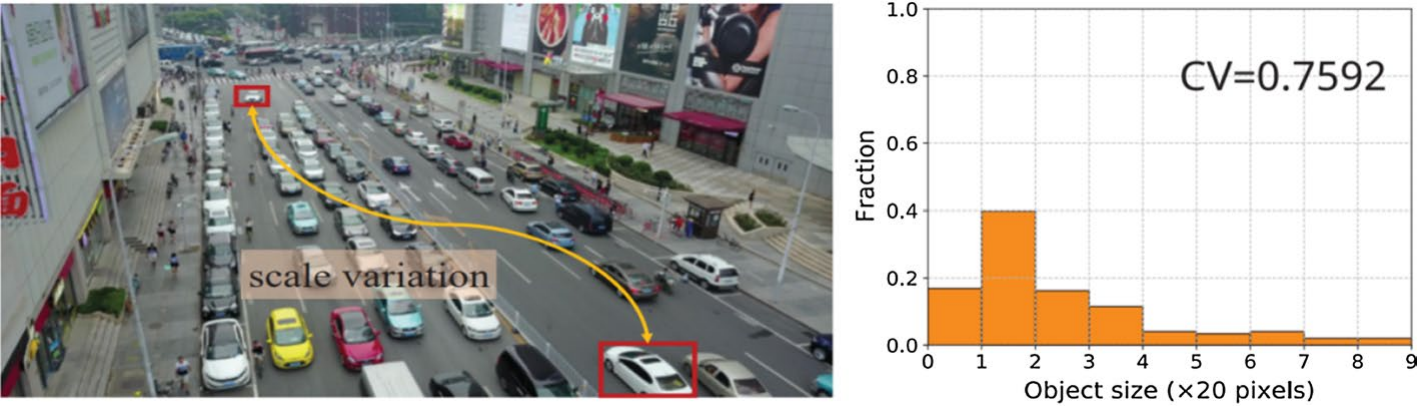
Example of scale variation and the scale (object size) distribution of the VisDrone2019 dataset objects in pixels [207]

One pervasive challenge in the scale invariant object detection is large scale variance across object instances, and particularly, detecting small objects are more challenging than medium and large-scale objects. As per MS COCO definition [206], Objects with size less than 32 × 32 pixels are small, size between 32 × 32 to 96 × 96 pixels are considered as medium and objects with size greater than 96 × 96 pixels are large objects (Table 2). On the one hand, small objects in MS COCO dataset accounts for only 1.23% of total object area, on the other hand, medium and large-scale objects are over 98% of object area. Object detection algorithms should be able to detect both small objects as well as medium and large objects. Detecting small objects are essential in many real-world applications. For instance, detecting distant or small objects in the high-resolution driving scene images captured from cars is essential for achieving autonomous driving. Many distant objects, such as traffic lights or cars, are imperceptible as shown in Fig. 26. Haoyue et al. [207] measure the extent of scale variation using the coefficient of variation (CV), determined as the ratio of the standard deviation to the mean of the object scale. The bigger the CV, the more complicated the problem of scale variation.

**Table 2:** The definitions and statistics of the small, medium, and large objects as MS COCO [206]

Object category	Spatial dimension		Object count %	Total object area %
	Minimum	Maximum		
Small	0 × 0	32 × 32	41.43	1.23
Medium	32 × 32	96 × 96	34.32	10.18
Large	96 × 96	∞ × ∞	24.24	88.59

There can be three reasons why detecting small objects are more complicated than larger one: 1. Small objects occupy a much smaller area, and consequently there exists lack of diversity where small objects are located in the image, 2. There are comparatively less images in the dataset containing small objects which may bias any object detection algorithm to concentrate more on medium and large-scale objects, and 3. The activations of small objects become smaller and smaller with each pooling layer in a standard convNet architecture as it progressively reduces the spatial size of an image.

To overcome the problem of scale imbalance, two different strategies based on GAN have been proposed in the literature. Commonly adopted strategy is to convert low resolution small object features into high resolution features [208] using GAN. Diversity of the small object locations in the images are enhanced by copy-pasting small object instances several times in each image through adversarial processes [209].

**Fig. 27:**
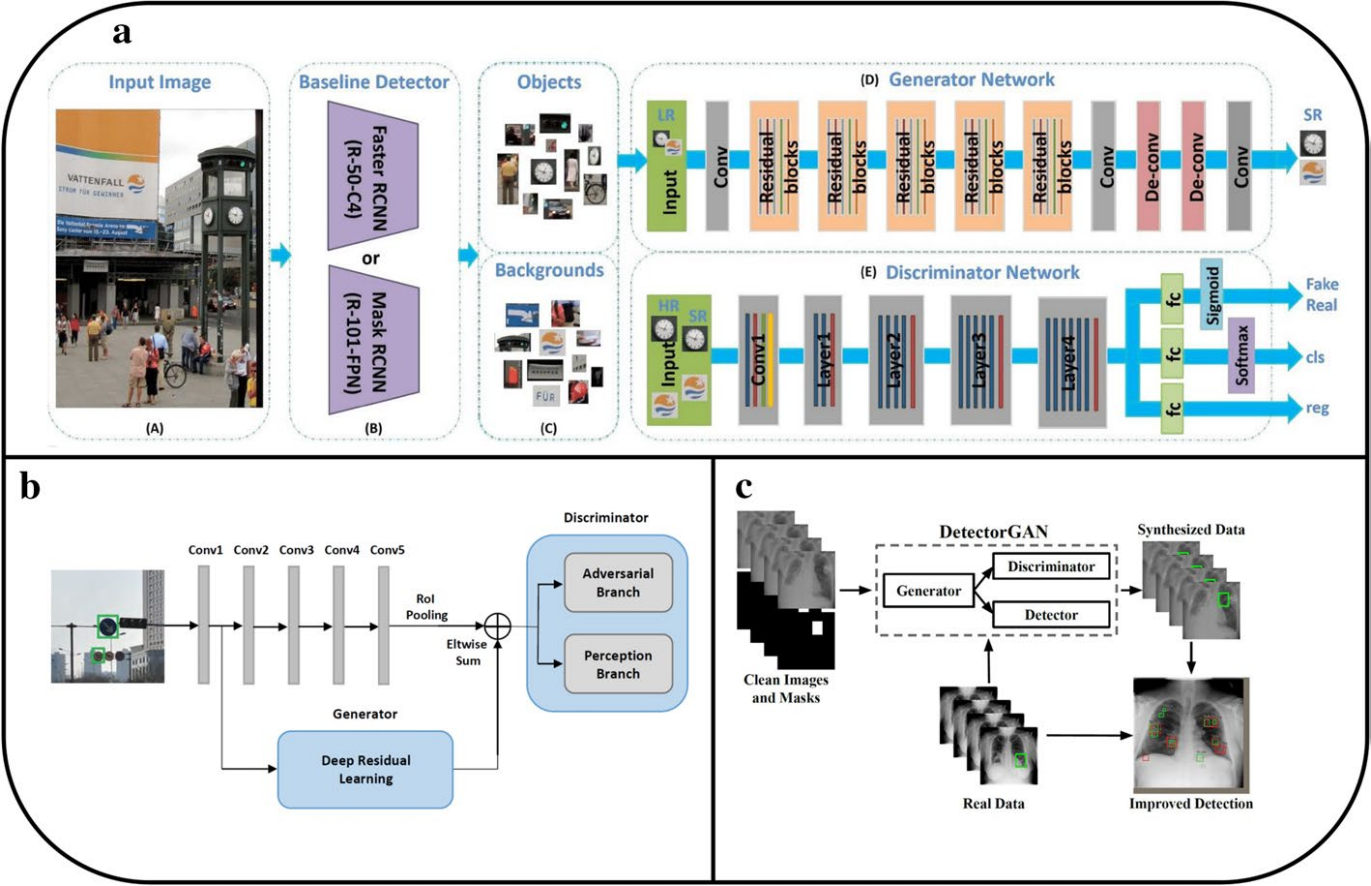
Architecture diagram of (**a**) SOD-MTGAN [213] (**b**) Perceptual GAN [208] and (**c**) Detector GAN [209]

Li et al. [208] utilized a GAN framework that transforms poor representation of small-scale objects to super-resolved large objects. The generator attempts to generate super resolution features for the small objects. The discriminator in this framework is decomposed into two branches, namely, a perceptual branch and an adversarial branch. An adversarial branch is trained to discriminate between real large-scale objects and generated super resolution objects while a perceptual branch helps to make sure that the generated super-resolved object is useful for the detection (Fig. 27b). They tested the effectiveness of this framework on Tsinghua-Tencent 100 k dataset [210], PASCALVOC dataset [211] and Caltech pedestrian benchmark [212].On the PASCAL VOC 2007 dataset [211], The Average precision (AP) of small objects such as plant, chair, bottle and boat increased by 10%, 15.1%, 21.9% and 10% respectively, compared with Faster-RCNN.

Bai et al. [213] used baseline detectors such as Faster RCNN [36], Mask RCNN [214] to crop an input image into smaller regions (generate ROIs) and then use generator network to reconstruct up-scaled version (super resolved) of cropped regions, while the discriminator perform multiple tasks that discriminates the real from the high resolution generated images, perform classification and regress the bounding box co-ordinates (object location) simultaneously (Fig. 27a).

Lanlan Liu et al. [209] proposed a Detector GAN that combines and optimizes both GANs and object detector together. The generator is trained with both adversarial and training loss, which generates multiple small objects in an image that are hard to detect by the detector and hence enhance the robustness of the detector (Fig. 27c).

#### Imbalance due to occlusions and deformations

**Fig. 28:**
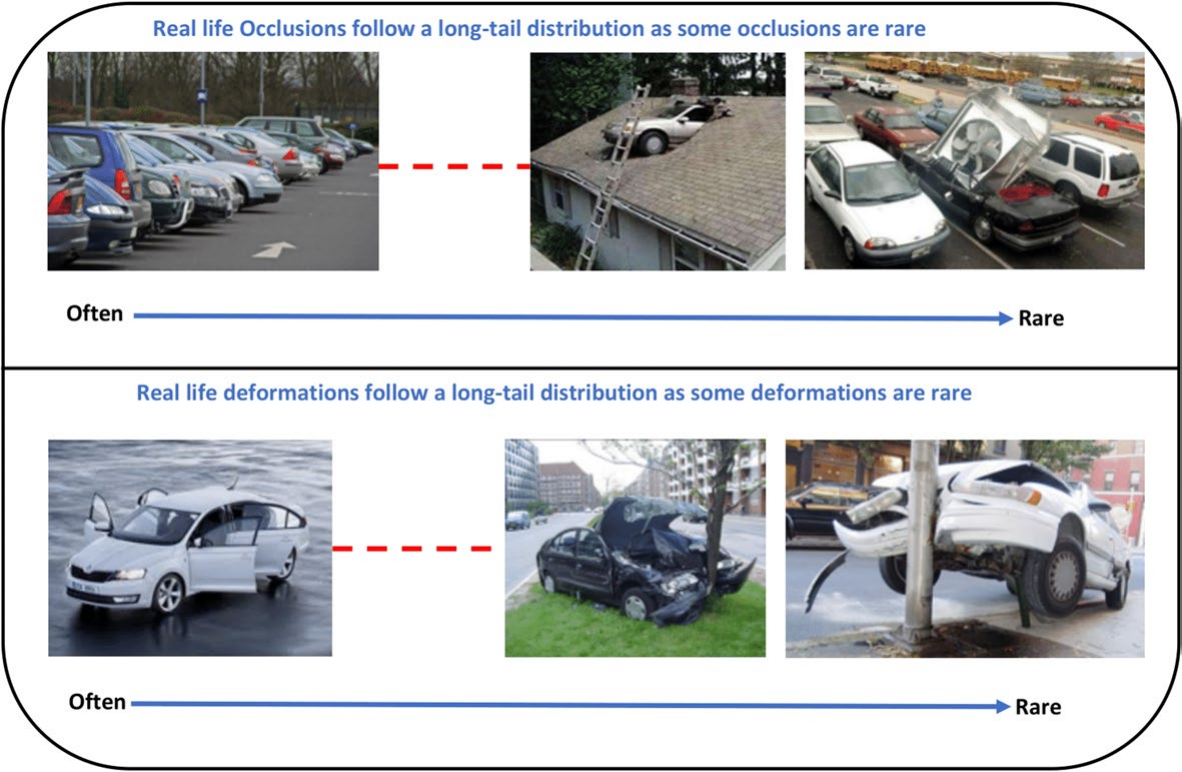
Imbalanced distribution of occluded, partially occluded and heavily occluded objects in VisDrone-DET2018 dataset [215]

**Fig. 29:**
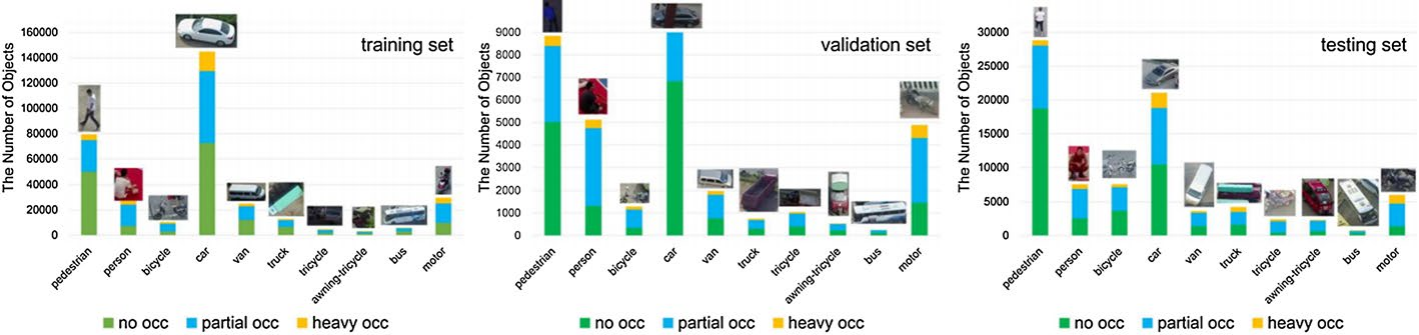
Illustration of real world occlusions and deformations provided by Wang et al. [216]

Like the object scale imbalance, occluded and deformed objects in the images follow a skewed distribution. For instance, occlusion from other cars due to urban traffic or parking lots is more common than from an air conditioner as shown in Fig. 28. The performance object detection is often suffered from imbalance due to occluded and deformed objects. Zhu et al. [215] define occlusion ratio to measure the degree of occlusion, determined as the fraction of pixels being occluded. As per VisDrone-DET2018 dataset [215], objects with occlusion ratio greater than 50% are heavy occlusion, ratio between 1 to 50% are considered as partial occlusion and objects with 0% occlusion ratio are categorized as no occlusion. The bar chart below (Fig. 29) depicts the imbalanced distributions of occluded, partially occluded and heavily occluded objects in VisDrone-DET2018 dataset [215].

One way to build the robust object detector invariance to occlusion and deformation is to generate realistic images of these rare occurrences using GANs, and then train the object detector with the generated images. Adversarial object detection could be another interesting way to generate all possible occlusions or deformations on the feature maps that make recognition hard. The object detector is simultaneously trained to overcome the difficulties imposed by the adversarial task.

**Fig. 30:**
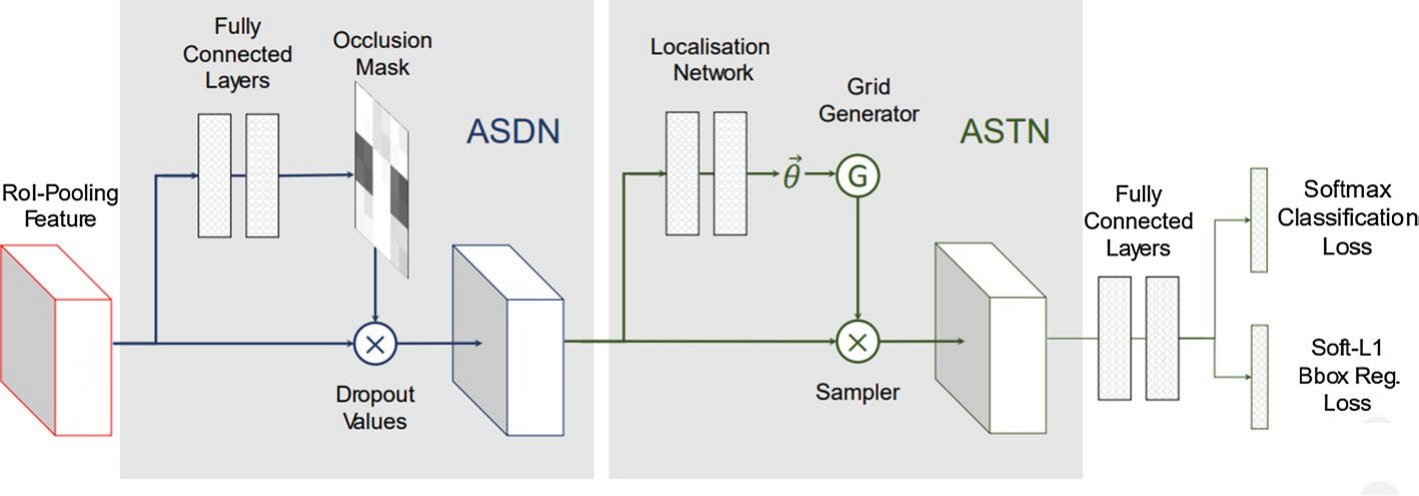
Architecture diagram to generate hard examples for training [216]

Wang et al. [216] utilized the adversarial spatial dropout to simulate all kinds of rare deformations and occlusions on the feature maps that are hard for the object detector to detect. Unlike traditional methods [49] that add occlusions on foreground objects in pixel space, they focused on feature space. Their architecture (Fig. 30) comprised of two networks: Adversarial Spatial Dropout Network (ASDN) and Adversarial Spatial Transformer Network (ASTN) to create occlusion and deformation respectively. On VOC2007 and VOC2012 datasets, this architecture achieved an increase in mean Average Precision (mAP) of 2.3% and 2.6% respectively compared to the Fast-RCNN [36].

Inspired by this architecture, Chen et al. [217]. proposed Adversarial Occlusion Aware Face Detection (AOFD) to overcome the problem of limited occluded face image in training dataset. As opposed to cropping or erasing, Dwibedi et al. [218]. utilized GAN to insert new objects on the images by cut and paste. This method can be extended by inserting occluded and deformed objects on the training images.

Taking full advantage of GANs and combining them into different ConvNet architectures is a recent trend in object detection. These kinds of architectures are often called a three-player GAN. In an attempt to improve performance of detection and classification, three-player GAN only generates hard-to-classify samples. Particularly, the use of faster R-CNN with GANs has improved the state of-the-art benchmarks. Testing the performance of different combinations in comparison to current state of-the-art models is an interesting area for future work.

#### Foreground–background object class imbalance

Both single stage and two stage object detection algorithms evaluate multiple regions in an image during the training stage. But only a few regions contain foreground (positive), the rest are background (negative). Many of the background examples are easy to classify and offer an uninformative training signal. Just a few background examples provide rich information for training. The imbalance between foreground (objects) and easily classified background overwhelms cross entropy loss and gradients from converging. Some form of hard sampling is a commonly used method by the object detection algorithms to account for this imbalance. The most straightforward and simple hard sampling method is uniform random sampling that randomly selects a subset of negative and positive examples (uniformly distributed) for evaluation. Hard negative mining is another hard sampling method that selects hard samples as negative examples instead of random selection to improve the detection performance.

**Fig. 31:**
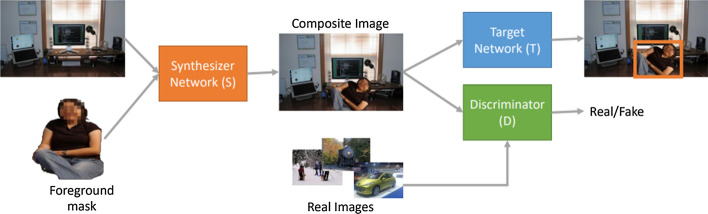
Pipeline of task aware image synthesis used by Tripathi et al. [219]

Unlike hard sampling methods, GAN addresses the problem of foreground background imbalance by directly injecting hard positive and negative synthetic examples into the training dataset. Task aware data synthesis proposed by Tripathi et al. [219]. uses GAN based approach to generate hard positive examples that improve the detectors classification accuracy. Their architecture utilizes three competing networks (Fig. 31): a synthesizer (S), a discriminator (D) and the target network (T).Given a background image and a hard-positive foreground mask, synthesizer aims to optimally paste foreground mask onto the background image to produce a realistic image that can fool both the target and discriminator networks. The discriminator network provides necessary feedback to the synthesizer which ensures the realism of the generated composite image. The target network is a pre-trained object detector such as SSD and faster R-CNN. On the VOC person detection dataset, this architecture achieved a performance improvement of up to 2.7%.

Wang et al. [220] presented an interesting idea of object detection via progressive and selective instance-switching (PSIS). Given a pair of training images, PSIS synthesizes a new pair of images by swapping objects of the same class between an original pair of images by also considering scale and shape information of the objects. Generating more training images by swapping objects of low-performing classes improves overall detection accuracy.

Gene-GAN [221] proposed by Zhou et al. employ an encoder and a decoder architecture to replace an object in an image with a different object from a second image. Given an image, Encoder decomposes it into the background and object feature vectors, while decoder reconstructs a new image by transplanting an encoded object to it.

### Pixel level imbalances in segmentation

#### Pixel-wise class imbalance

GANs are being employed to solve pixel level class imbalance problem in segmentation tasks that have a negative influence on segmentation accuracy. The use of image to image translation GANs for a pixel-level augmentation on segmentation tasks was tested by Liu et al. [222]. Particularly, they used Pix2pix HD GAN [143] to translate semantic label maps to realistic images. Semantic object labels from the original dataset such as street, car, pedestrian etc. are recombined to synthesize new label maps which can balance the semantic label distribution. Then the new balanced label maps are translated to realistic images by Pix2pix HD GAN. To further understand the effectiveness of this method, a study was conducted by balancing one to many label classes on original label maps. On the Cityscapes dataset [57] this resulted in an improved mean accuracy of a specific class up to 5.5% and the average overall segmentation accuracy up to 2%.

**Fig. 32:**
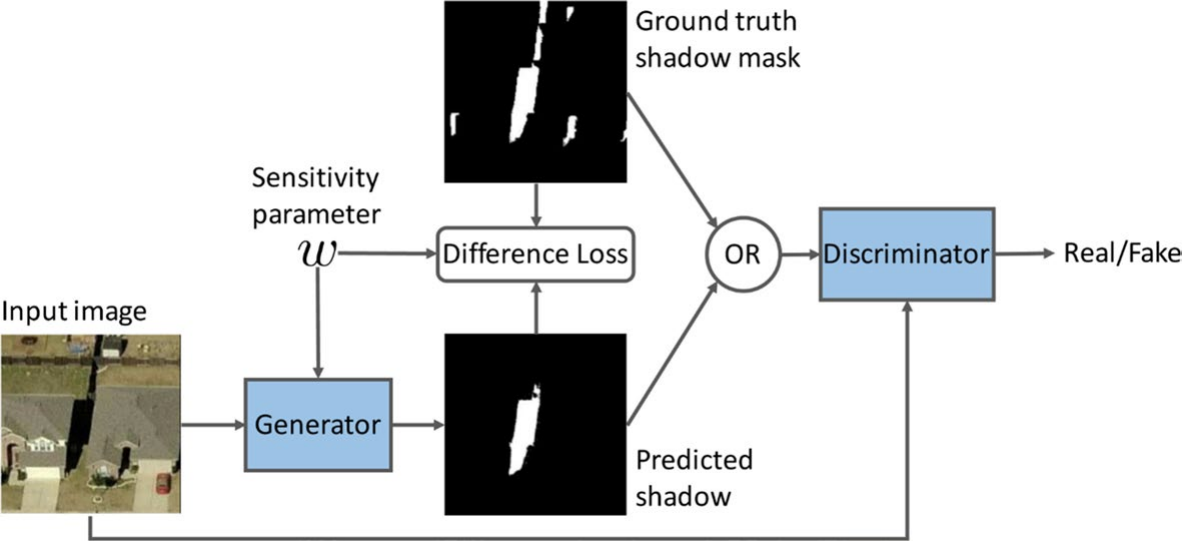
Illustration of Sensitivity conditional GAN [223]

Shadow detection is a segmentation problem in which there are substantially lesser shadow pixels than non-shadow pixels in training images. Nguyen et al. [223] presented Sensitivity conditional GAN (ScGAN), an extension of cGAN [118], tailored to tackle the challenging problem of pixel-level imbalance. To balance shadow and non-shadow pixel imbalance during training process, Sensitivity parameter $W$ is introduced in ScGAN that controls how much to penalize the false positive prediction. Notably, the Sensitivity parameter $W$ is made tunable by allowing it to interact with the generator in addition to loss function (Fig. 32). ScGAN achieved up to 17% error reduction on UCF [224] and SBU [225] dataset with respect to the previous state-of-the-art model.

Voxel GAN architecture proposed by Rezaei et al. [226] is a 3D GAN model to address the pixel level imbalance problem in the brain tumor segmentation task as the majority of the pixels belongs to the healthy region and only few pixels belongs to tumor region. Voxel GAN is made of 3D segmentor network to learn generating segmentation labels from 3D MRIs, and a discriminative network to differentiate generated segmentation labels from real labels. The segmentor and discriminator are trained by mix of adversarial loss with weighted $\ell 1$ loss and weighted categorical cross-entropy loss to reduce the negative impact of pixel imbalance.

Similar to this work, Rezaei et al. [227] used similar loss function by mixing adversarial loss and weighted categorical accuracy loss to handle imbalanced training dataset of whole heart segmentation tasks. Balancing through ensemble learning by combining two discriminators to improve their generalization ability of the GAN was tested by Rezaei et al. [228] in medical image semantic segmentation task. One discriminator classifies whether the generated segmentation label is real or fake. Another discriminator is trained to predict false positives and false negatives. Final segmentation mask is generated through adding the false negatives and removing the false positives predicted by this discriminator.

#### Imbalance due to occlusions in segmentation

GANs are also very efficient in segmentation of natural settings with severe occlusion and large-scale changes [229]. Sa et al. [230] describe that occlusion is a key challenge in segmenting dense scenes. Objects in dense scenes often occlude each other, which lead to severe information loss. In many cases, segmentation algorithms cannot infer the appearance of the objects beyond their visible parts, which may prevent it from making accurate decisions if a person purposely covers the face. GANs offer a new way to generate the invisible parts of objects, i.e., learns to complete the appearance of occluded objects.

**Fig. 33:**
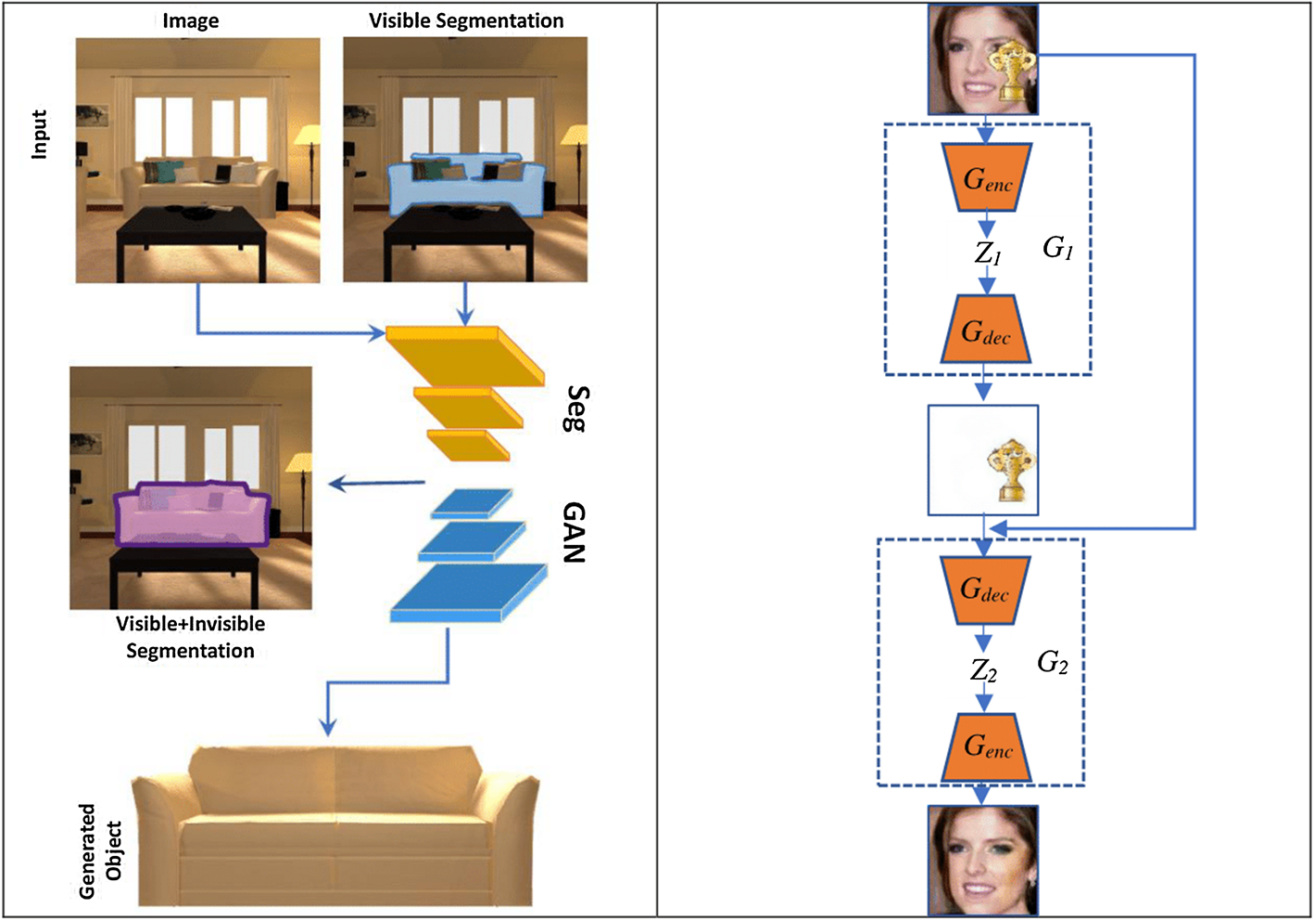
Illustration of SeGAN [231] (left) and Occlusion-Aware GAN [232] (right)

SeGAN [231], developed by Ehsani et al., is an interesting framework to segment the invisible part of the object and then generate the appearance by painting the invisible parts. The proposed framework uses a segmentor, a generator, and a discriminator to combine segmentation and generation tasks (Fig. 33). The segmentor takes an image and segmentation mask of the visible region of an object as an input, and then predicts an intermediate mask of the entire occluded object. The generator and discriminator are trained to generate an object image in which the invisible regions of the object are reconstructed.

Dong et al. [232] proposed a two stage model, named Occlusion-Aware GAN (OA-GAN), to remove arbitrary facial occlusions, e.g., faces with mask, microphone, cigarette, etc. OA-GAN is equipped with two GANs: The first GAN $G_{1}$ is designed to disentangle the occlusion, and the second GAN $G_{2}$ is trained to generate the occlusion free images given the generated occlusions.

## Discussion

To provide a detailed overview and better comparison of various studies for imbalances in computer vision, the surveyed works have been summarized in Table 3.

**Table 3:** Comparative summary of GANs for the problem of imbalances in computer vision

Category	Imbalance type	Study	Application
Binary classification	Inter class imbalance	DCGAN [153]	Malaria disease classification
	Inter class imbalance	SDGAN [154]	Industrial defect classification
	Inter class imbalance	BAGAN [155]	Image classification
	Inter class imbalance	CiGAN [156]	Mammogram classification
	Inter class imbalance	CycleGAN [157]	Mammogram classification
	Inter class imbalance	DCGAN [233]	Mammogram classification
	Inter class imbalance	CovidGAN [159]	Covid19 classification
	Intra class imbalance	Clustering + GAN [163]	Imbalanced intra class classification
	Intra class imbalance	Semantically decomposed GAN [234]	Imbalanced intra class classification
	Intra class imbalance	VAE + GAN [115]	Facial Attribute editing
	Intra class imbalance	AttGAN [64]	Facial Attribute editing
	Intra class imbalance	IcGAN [65]	Facial Attribute editing
	Intra class imbalance	ResAttr-GAN [66]	Facial Attribute editing
	Intra class imbalance	ARU-net [170]	Facial Attribute editing
	Intra class imbalance	SaGAN [171]	Facial Attribute editing
	Intra class imbalance	PN-GAN [176]	Person reidentification
	Intra class imbalance	PTGAN [177]	Person reidentification
	Intra class imbalance	CycleGAN [178]	Person reidentification
	Intra class imbalance	SPGAN [179]	Person reidentification
	Intra class imbalance	FDGAN [180]	Person reidentification
	Intra class imbalance	Cross view GAN [182]	Vehicle reidentification
	Intra class imbalance	DCGAN [183]	Vehicle reidentification
	Intra class imbalance	F-CGAN [184]	Fine grained classification
	Intra class imbalance	DCGAN + Fine grained Classifier [188]	Fine grained classification
	Intra class imbalance	General-to-Detailed GAN [190]	Fine grained classification
Multi class classification	Few minority-many majority class imbalance	Cycle GAN [197]	Emotion classification
	Few minority-many majority class imbalance	DCGAN [198]	Weather classification
	Few minority-many majority class imbalance	DCGAN + Ensemble learning [199]	Weather classification
	Few minority-many majority class imbalance	DCGAN [193]	Chest pathology classification
	Few minority-many majority class imbalance	DCGAN [200]	liver lesion classification
	Many majority- Few minority class imbalance	DCGAN [201]	Skin lesion classification
	Many majority- Many minority class imbalance	Cycle-GAN [192]	Plant disease classification
	Many majority- Many minority class imbalance	WGAN-GP [203]	Multi class classification
	Many majority- Many minority class imbalance	CE-GAN [205]	Multi class classification
Object detection	Object Scale imbalance	Perceptual GAN [208]	Traffic sign detection
	Object Scale imbalance	SOD-MTGAN [213]	Small object detection system
	Object Scale imbalance	Detector GAN [209]	Pedestrian and disease detection
	Imbalance due to occlusions and deformations	Adversarial-Fast-RCNN [216]	Occluded object detection
	Imbalance due to occlusions and deformations	Adversarial Occlusion-aware Face Detector [217]	Occluded face detection
	Imbalance due to occlusions and deformations	Cut-Paste GAN [218]	Occluded object detection
	Foreground Background object class imbalance	Task-aware synthetic data generation [219]	Object detection
	Foreground Background object class imbalance	Gene-GAN [221]	Object detection
	Foreground Background object class imbalance	PSIS [220]	Object detection
Segmentation	Pixel wise Imbalance	Sensitivity conditional GAN [118]	Shadow detection
	Pixel wise Imbalance	Pix2pix HD GAN [143]	Imbalanced pedestrian image segmentation
	Pixel wise Imbalance	Voxel GAN [226]	Brain tumor segmentation
	Pixel wise Imbalance	GAN + ensemble learning [228]	Medical image semantic segmentation
	Pixel wise Imbalance	GAN + Weighted categorical loss [227]	Heart image segmentation
	Imbalance due to occlusions	SeGAN[231]	Invisible part generation and Segmentation
	Imbalance due to occlusions	Occlusion-Aware GAN [232]	Occlusion free image generation

GANs based methods that address the imbalance problem in classification tasks aim to increase the classification accuracy for the minority classes. Many of these methods use image-to-image translation to generate minority class images from one of the majority classes, while others generate minority class images from the random noise vector. GANs based intelligent oversampling [197] method outperforms both traditional sampling and data augmentation methods in classifying imbalanced image data. However, it is not clear how much synthetic images must be blended with original images to achieve the maximum performance of the classifiers. Additionally, synthetic images would lead to additional noise to the original training dataset if the quality of the synthesized images is poor. Therefore, most of the surveyed methods in GANs based intelligent oversampling methods [197] focused mainly on balancing distribution as well as improving quality of the generated images.

Image-to-image translation [138] methods used for inter-class imbalance problem cannot be extended to solve intra-class imbalance as it is difficult to acquire image datasets with detailed labels. The interesting way to solve this problem is to employ clustering techniques in the feature space of the GANs to divide the images into different groups for automatic pattern recognition in the dataset. Improving the performance of the clustering techniques that clearly find the difference among clusters, is an area of future work.

GANs and encoder network hybrid models have a good potential to address intra class imbalance problem in face recognition and re-identification tasks. The key idea of these models is to work on latent code space rather than the pixel space. This is because for manipulating a fine grained image category, e.g., hair color, the latent code representation will operate only on that single latent code (hair color), whereas the pixel space will edit every single pixel in an image.

The fascinating approaches to use GANs for the problem of object level imbalances in object detection tasks fall into two general categories: 1. Generating more rare examples as intelligent oversampling used for class imbalance. These generated rare examples are introduced into the training dataset to address imbalance problems. 2. Learn an adversary in combination with original object detection algorithms. This adversary modifies the features to solve imbalance problems instead of generating examples in pixel space. i.e., to generate hard-to-detect samples by performing feature space manipulations.

The capability of super-resolution GANs are being used to up-sample small blurred objects into fine-scale ones and to recover detailed spatial information for accurate small object detection. This technique combines super-resolution GANs with object detection algorithms to solve the imbalances due to object size. The power of adversarial process is being used to increase the diversity of the small object locations in the images by copy-pasting small object instances several times at different locations.

Making the best use of GANs and combining them into U-Net architectures is an interesting way to solve pixel level imbalances in segmentation tasks. These architectures often use a weighted loss function to mitigate the pixel level imbalances. Combination of image in painting GANs with U-Net architectures has the great potential use in segmenting hidden objects. This technique is not only efficient in segmentation tasks, but also to infer the appearance of the objects beyond their visible parts. Overall, combining different deep learning models with adversarial process can provide a way to solve many other open problems in the computer vision field.

## Future work

Even though GANs can be used as an effective way to unlock additional information from a dataset, the synthetic images generated by GANs cannot replace the real images completely. However, a blend of different proportions of real and GANs generated images are extremely useful to improve the diversity of the training samples and increase performance of the classifiers. Our future work intends to study the influences of blending different propositions of GANs generated images and real images on the classification performance. There are a very limited number of comparative studies that compare effectiveness of using GAN based synthetic images with other traditional methods for intra-class imbalances. We also intend to conduct the comparative study in order to validate the effectiveness of using synthetic images for intra class imbalances.

Inflating the size of the dataset brings another problem: One of the most significant limitations in computer vision experiments is computational resources. Sophisticated computer vision models trained on inflated dataset can perform complex tasks, the problem however is, how do we deploy such massive architecture on edge devices for instant usage. Handling this problem using knowledge distillation is non-trivial and an active field of research. Knowledge distillation is model compression technique in which a smaller network is trained with the help of the sophisticated pretrained model to achieve the similar accuracy. This training process is often referred to as "teacher-student”, where the sophisticated pretrained model is the teacher and the smaller network is the student. Wang et al. [235] combine GANs and knowledge distillation to improve the efficiency of the student network in object detection. Similar to this work, we will attempt to further implement GANs and knowledge distillation combinations to other computer visions tasks.

As research on GANs are developing and maturing, assessment of performance has become essential. Evaluation metrics helps to quantitatively measure how well GANs models are performing, also to assess the relative performance of GANs. Very often the performance of GANs is measured by the manual inspection of the visual fidelity of generated images. However, the manual inspection is cumbersome, subjective, time-consuming, and sometimes misleading. Lack of universal evaluation metrics can impede the development of GANs. Introducing new performance measures to evaluate both diversity and fidelity of generated images is a very important area for future work.

Manually designing GANs architecture for a given task is time-consuming and sometimes has a tendency of errors. This drawback has led researchers to move on to the next stage of automating GANs architecture in the form of neural architecture search (NAS). Another interesting area of further research is to use meta-heuristic search algorithms that assist architectural search and find optimal GANs architecture which outperforms human created GANs models.

Achieving equilibrium between the generator and discriminator of the GANs can take a long time relative to other deep neural networks. Distributed training of GAN through parallelization and cluster computing is another important area of future work to cut down the training time.

Most of the applications of the GANs so far have been for creating synthetic images. GANs are not limited to the visual domain and can be also applied to non-visual applications. For example, Paganini et al. [236] used GANs to predict the outcome of high energy particle physics experiments. Instead of using explicit Monte Carlo simulation of the real physics of every step, the GANs learn by example what outcome is likely to occur in each situation. The GANs reduce the computational cost of high energy particle simulation, enough to save millions of dollars' worth of supercomputer time. We believe that the invention of new applications using this powerful tool will be continued in the future.

## Conclusion

This paper surveys various GANs architectures that have been used for addressing the different imbalance problems in computer vision tasks. In this survey, we first provided detailed background information on deep generative models and GAN variants from the architecture, algorithm, and training tricks perspective. In order to present a clear roadmap of various imbalance problems in computer vision tasks, we introduced taxonomy of the imbalance problems. Following the proposed taxonomy, we discussed each type of problems separately in detail and presented the GANs based solutions with important features of each approach and their architectures. We focused mainly on the real-world applications where GAN based synthetic images are used to alleviate class imbalance. In addition to the thorough discussion on the imbalance problems and their solutions, we addressed many open issues that are crucial for computer vision applications.

Synthetic but realistic images generated using the methods discussed in this survey have the potential to mitigate the class imbalance problem while preserving the extrinsic distribution. Many of the methods surveyed in this paper tackled the highly complex imbalances by combining GANs architecture with different other deep learning frameworks. Specifically, the use of autoencoders with GANs has offered an effective way to perform feature space manipulations instead of complex pixel space operations.

Synthetic images generated by GANs cannot be used as the complete replacement for real datasets. However, the blend of real and GANs generated images have enormous potential to increase the performance of the deep learning model. Looking into the future, GAN-related research in image as well as non-image data domains to address the problem of imbalances and limited training dataset would continue to expand. We conclude that the future of GANs is promising and there are clearly a lot of opportunities for further research and applications in many fields.

## Data Availability

Not applicable.

## Abbreviations

ConvNets: Convolutional neural networks
SMOTE: Synthetic minority oversampling technique
ADASYN: Adaptive synthetic sampling
IHM: Instance hardness measure
SSL: Semi-supervised learning
R-CNN: Region-based convolutional neural networks
RPN: Region proposal network
YOLO: You only look once
SSD: Singe shot detection
SNIP: Scale normalization for image pyramids
FPN: Feature pyramid networks
RNN: Recurrent neural networks
LSTM: Long short-term memory
PCA: Principle component analysis
MADE: Masked autoencoder density estimator
ARs: Autoregressive models
FVBNs: Fully visible belief networks
RGB: Red Green blue
NADE: Neural autoregressive density estimator
MADE: Masked autoencoder density estimator
VAEs: Variational auto encoders
CVAE: Conditional variational auto encoders
DC-IGN: Deep convolutional inverse graphics network
IWVAE: Importance weighted Variational Auto Encoders
VQ-VAEs: Vector quantized variational auto encoders
DRAW: Deep recurrent attentive writer
EMD: Earth mover Distance
TTUR: Two time-scale update rule
DDSM: Digital database for screening mammography
ARU-net: Adversarially regularized U-net
AMN: Attribute manipulation network
SiaNet: Siamese network
CV: Coefficient of variation
AP: Average precision
ASTN: Adversarial spatial transformer network
ASDN: Adversarial spatial dropout network
mAP: Mean average precision
AOFD: Adversarial occlusion aware face detection
PSIS: Progressive and selective instance-switching
ADAM: Adaptive moment estimation optimizer
ReLU: Rectified linear unit
GANs: Generative adversarial neural networks
cGAN: Conditional generative adversarial network
ACGAN: Auxiliary classifier generative adversarial network
VACGAN: Versatile Auxiliary classifier generative adversarial network
InfoGAN: Information maximizing generative adversarial network
SCGAN: Similarity constraint generative adversarial network
DCGAN: Deep convolutional generative adversarial network
ProGAN: Progressive growing of generative adversarial network
LAPGAN: Laplacian generative adversarial network
GRAN: Generative recurrent adversarial networks
D2GAN: Dual discriminator generative adversarial network
MADGAN: Multi-agent diverse generative adversarial network
CoGAN: Coupled generative adversarial network
DEGAN: Decoder encoder generative adversarial network
VAEGAN: Variational autoencoder generative adversarial network
AAE: Adversarial autoencoders
ALI: Adversarially learned inference
BiGAN: Bidirectional generative adversarial network
SRGAN: Super-resolution generative adversarial network
SAGAN: Self-attention generative adversarial network
WGAN: Wasserstein generative adversarial network
WGAN-GP: Wasserstein generative adversarial network with gradient penalty
LSGAN: Least squares generative adversarial network
EBGAN: Energy based generative adversarial network
BEGAN: Boundary equilibrium generative adversarial network
SD-GAN: Surface defect-generative adversarial network
BAGAN: Balancing generative adversarial network
ciGAN: Conditional infilling generative adversarial network
IcGAN: Invertible conditional generative adversarial network
PNGAN: Pose-normalized generative adversarial network
PTGAN: Person transfer generative adversarial network
SPGAN: Similarity preserving cycle consistent generative adversarial network
FD-GAN: Feature distilling generative adversarial network
F-CGAN: Fine grained conditional GAN
CE-GAN: Class expert generative adversarial network
ScGAN: Sensitivity conditional generative adversarial network
OAGAN: Occlusion-aware generative adversarial network
